# Quantitative Estimation of Oxidative Stress in Cancer Tissue Cells Through Gene Expression Data Analyses

**DOI:** 10.3389/fgene.2020.00494

**Published:** 2020-05-19

**Authors:** Liyang Liu, Haining Cui, Ying Xu

**Affiliations:** ^1^College of Physics, Jilin University, Changchun, China; ^2^Department of Biochemistry and Molecular Biology, Institute of Bioinformatics, The University of Georgia, Athens, GA, United States; ^3^Cancer Systems Biology Center, The China-Japan Union Hospital, Jilin University, Changchun, China

**Keywords:** oxidative stress, genomic mutation, transcriptomic data, cancer, TCGA data analysis, computational prediction

## Abstract

Quantitative assessment of the intracellular oxidative stress level is a very important problem since it is the basis for elucidation of the fundamental causes of metabolic changes in diseased human cells, particularly cancer. However, the problem proves to be very challenging to solve *in vivo* because of the complex nature of the problem. Here a computational method is presented for predicting the quantitative level of the intracellular oxidative stress in cancer tissue cells. The basic premise of the predictor is that the genomic mutation level is strongly associated with the intracellular oxidative stress level. Based on this, a statistical analysis is conducted to identify a set of enzyme-encoding genes, whose combined expression levels can well explain the mutation rates in individual cancer tissues in the TCGA database. We have assessed the validity of the predictor by assessing it against genes that are known to have anti-oxidative functions for specific types of oxidative stressors. Then the applications of the predictor are conducted to illustrate its utility.

## Introduction

Oxidative stress has long been recognized as a cellular stress associated with cancer formation and development (Cerutti and Trump, 1993; Wangpaichitr et al., 2009; Bellezza et al., 2010; Chan et al., 2010; Conti et al., 2010; Dayem et al., 2010; Gibellini et al., 2010; Henkler et al., 2010; Karlenius and Tonissen, 2010; Massi et al., 2010; Ortega et al., 2010; Pizzimenti et al., 2010; Reuter et al., 2010; Schultz et al., 2010; Soory, 2010; Sun et al., 2010; Valle et al., 2010; Gorrini et al., 2013; Sosa et al., 2013; Leone et al., 2017). It refers to the gap between the intracellular oxidizing power and the anti-oxidation capacity in a human cell. Numerous authors have pointed out that oxidative stress may be responsible for the induction of a variety of altered metabolisms in cancer, which include (i) considerably increased lipid metabolism (Santos and Schulze, 2012; Zhang and Du, 2012; Alfaradhi et al., 2014; Zhang et al., 2014), (ii) altered metabolisms of sulfur-containing amino acids (Schulz et al., 2000; Zhang et al., 2012; Campbell et al., 2016; Martínez et al., 2017), (iii) reprogrammed sugar metabolisms (Takeyama et al., 2000; Wu et al., 2009; Dewald et al., 2016), among other reprogrammed metabolisms. Increased genomic mutations in cancer have also been attributed to oxidative stress (Limoli and Giedzinski, 2003; Doudican et al., 2005; Slane et al., 2006; Xu et al., 2014; Fitzgerald et al., 2017; Markkanen, 2017; Rao et al., 2017; Tubbs and Nussenzweig, 2017). However, it has proven to be very challenging to accurately estimate the level of intracellular oxidative stress (Selvaraj et al., 2002; Farah, 2005). Because existing techniques are mostly designed to detect quantitatively specific oxidizing molecular species (e.g., H2O_2_ and O_2_) rather than detecting the overall level of oxidative stress. Computational techniques could potentially play a key role in estimating the quantitative level of oxidative stress.

Unlike hypoxia or acid-base imbalance whose related stress can be measured/estimated in terms of the concentration of one or a few molecular species such as O_2_ or H+, there are many types of oxidizing molecules such as reactive oxygen species (Barash et al., 2010; Reczek and Chandel, 2017), reactive nitrogen species (Fionda et al., 2016; Kruk and Aboul-Enein, 2017), reactive lipid species (Higdon et al., 2012; Graham, 2016) among others. Furthermore, to estimate the stress level, it also requires to know the total cellular reducing capacity. This is also challenging since human cells not only have a basic set of anti-oxidation (or reducing) capacities consisting of (1) glutathione and associated enzymes (e.g., glutathione transferase and peroxidase), (2) vitamin A, C, E and derivatives like beta-carotene, and (3) anti-oxidation enzymes like *SOD (superoxide dismutase), PRX (periaxin)*, and *TRX (thioredoxin)* (Chang et al., 2008; Mantovani et al., 2010), but also rely on anti-oxidation capacities of various other molecules including some enzymes and fatty acids. Among them, the main functions of the enzymes may not be for anti-oxidation (Osmundsen et al., 1982; Wieczorek et al., 2017), while the anti-oxidative properties of fatty acids have long been well-established (Richard et al., 2008; Freitas et al., 2017). All these make it very difficult to pin down on what molecular species should be used when assessing the level of oxidative stress.

A few proposals have been made regarding possible biomarkers for intracellular level of oxidative stress such as the carbonylation level (aldehydes and ketones) of proteins (Dalle-Donne et al., 2003; Hacişevki et al., 2012; Fernando et al., 2016), the level of oxidized low-density lipoprotein (Itabe, 2012; Osman et al., 2016), oxidized products of lipids such as *4-HNE (4-hydroxynonenal) and MDA (malondialdehyde)* (Niki, 2008; Halder and Bhattacharyya, 2014; Teppner et al., 2015), and protein thiols (Giustarini et al., 2012, 2017). There are two general issues with these biomarkers: (1) they tend to reflect the level of oxidation by specific oxidizing molecules; and (2) more importantly, they are not high-throughput, hence it is impractical for large-scale analyses, such as analyses of TCGA tissue samples (https://portal.gdc.cancer.gov/) to elucidate possible causes of specific metabolic changes in such tissues.

The goal here is to identify a set of genes whose mRNA expression levels can collectively reflect the overall level of intracellular oxidative stress present in a cancer tissue. The strategy is: (1) the somatic point-mutation rate in each cancer tissue sample is used as an indicator for the level of intracellular oxidative stress, an idea that has been well-established and applied (Doudican et al., 2005; Slane et al., 2006; Xu et al., 2014; Fitzgerald et al., 2017; Markkanen, 2017; Rao et al., 2017; Tubbs and Nussenzweig, 2017); (2) a few enzyme classes are selected, which are known to have anti-oxidation activities such as EC 3.1.-, EC 3.6.-, EC 2.4.-, and EC 2.7.- (Kato et al., 1995; Kong et al., 2007); and (3) a subset of genes are selected from these enzyme classes, whose combined expressions correlate strongly with the mutation rates in the matching genomes, determined through regression analyses. One assumption used in the analysis is that the oxidative stress levels in cytosol and nucleus are the same, which is reasonable knowing that the nuclear pores are large enough to allow most of the oxidizing molecules to go through freely between the two compartments; and increased mutation rates in cancer are known to be related to the nucleus oxidative stress level (Chung et al., 2014; Markkanen, 2017).

By applying this strategy to gene-expression data and matching genomic mutation data of cancer tissues of 14 cancer types in TCGA, representing all those with sufficiently large sample sizes, we have trained a predictor for the intracellular level of oxidative stress for each of the 14 cancer types. They are BLCA (bladder urothelial carcinoma), BRCA (breast invasive carcinoma), COAD (colon adenocarcinoma), ESCA (esophageal carcinoma), HNSC (head and neck squamous cell carcinoma), KICH (kidney renal papillary cell carcinoma), KIRC (kidney chromophobe), KIRP (kidney renal clear cell carcinoma), LICH (liver hepatocellular carcinoma), LUAD (lung squamous cell carcinoma), LUSC (lung adenocarcinoma), PRAD (prostate adenocarcinoma), STAD (stomach adenocarcinoma), and THCA (thyroid carcinoma). We have then validated the predictor against data with known oxidative stress related information. A key advantage in having a gene-expression data-based predictor is that RNA-seq data tend to be collected in general for cancer research; and such a predictor does not require a user to know the mutation rate distribution of the cancer type under study since such information is already encoded in the predictor for each cancer type.

## Results

### Genomic Mutation Profiles

**Figure 1:**
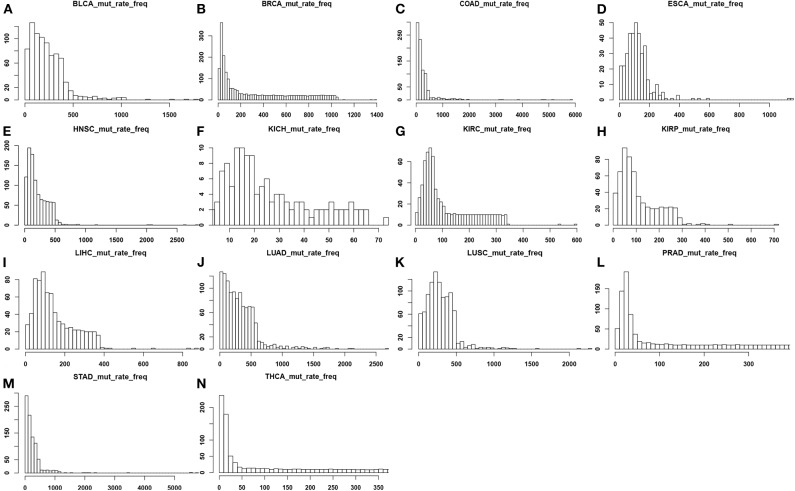
Distributions of the number of point mutations across all samples for each of the 14 cancer types, where the x-axis represents the mutation rate and the y-axis denotes the frequency of mutation rate across the tissue samples in each cancer type. **(A)** BLCA; **(B)** BRCA; **(C)** COAD; **(D)** ESCA; **(E)** HNSC; **(F)** KICH; **(G)** KIRC; **(H)** KIRP; **(I)** LIHC; **(J)** LUAD; **(K)** LUSC; **(L)** PRAD; **(M)** STAD; **(N)** THCA.

We have calculated and plotted the distribution of the number of point-mutations in coding regions per genome across all cancer genomes for each of the 14 cancer types, as shown in Figure 1. From the figure, LUAD has the highest average mutation rate at 347.20 per genome, and KICH has the lowest one at 19.75 per genome. The following lists the average numbers of the remaining 12 cancer types in the descending order: 288.95 mutations in LUSC, 272.02 in COAD, 257.50 in STAD, 234.71 in BLCA, 213.26 in HNSC, 124.59 in ESCA, 147.65 in LIHC, 303.75 in BRCA, 111.14 in KIPR, 113.94 in KIRC, 138.75 in PRAD, and 134.18 in THCA.

### Selection of Antioxidant Enzyme-Encoding Genes

Some classes of enzymes have long been known to have anti-oxidative functions as their main function such as the antioxidant enzymes mentioned earlier while some others have anti-oxidation as their secondary function such as cytochromes P450 and mitogen activated protein kinases (Limón-Pacheco and Gonsebatt, 2009). And yet, increasingly more proteins have been found to have antioxidant roles in addition to their main functions such as translocases (Tang et al., 2017), hydrolase (Liu et al., 2018), and hexokinase (Heneberg, 2019).

Based on such information, we have conducted a preliminary regression analysis of the mutation rates against the expression data of all the enzyme-encoding genes in the same samples for each of the 14 cancer types (with a Lasso penalty). Interestingly, genes that give rise to good regression results across all 14 cancer types generally fall into a small set of enzyme subclasses, particularly EC 3.- (hydrolases) and EC 2.- (transferases). Hence, we have conducted a second round of regression against expression data of genes only in these two EC classes.

From genes selected for each of the 14 regression models, we note the following: (1) in all 14 cancer types, genes in each regression model fall into exactly four sub-subclasses of EC 2.- and EC 3.-; (2) in five cancer types: BLCA, KIRC, KIRP, LUAD, and LUSC, all the genes fall into four sub-subclasses of EC 3.-; in three cancer types, BRCA, ESCA and KICH, all genes fall into four sub-subclasses of EC 2.-; and in the remaining six cancer types: COAD, HNSC, LIHC, PRAD, STAD, and THCA, all genes fall into two subclasses of EC 2.- and two subclasses of EC 3.-; (3) the two most commonly used EC 3.- subclasses are EC 3.4.21.- and EC 3.1.3.- while the two mostly used EC 2.- subclasses are EC 2.4.1- and EC 2.7.1.-. Table S1 gives the gene names selected in the regression model for each cancer type.

### Linear Regression Analyses

**Figure 2:**
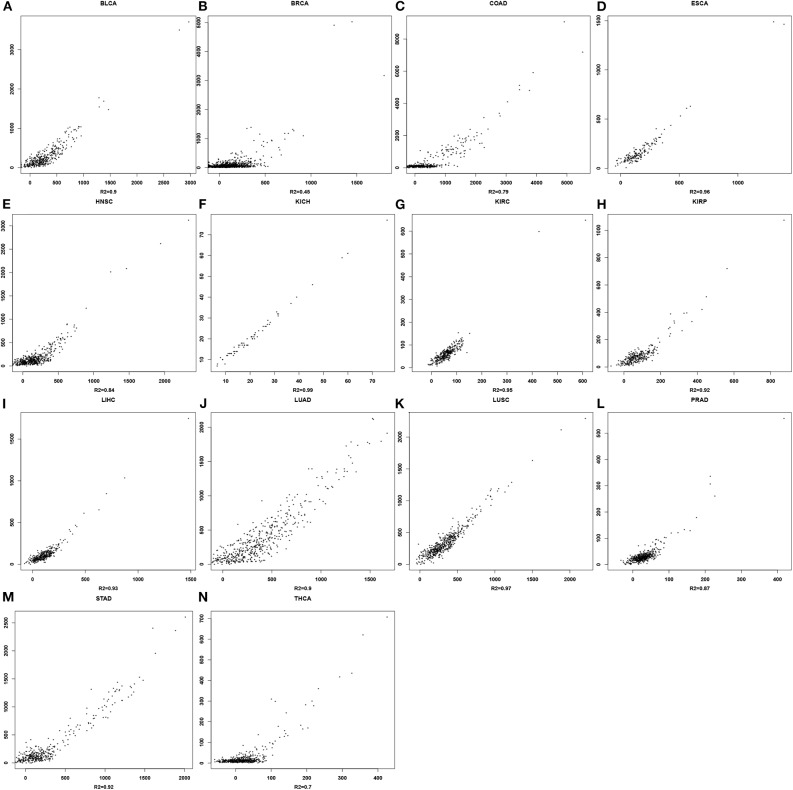
Scatter plots for mutation rates vs. predicted values in each of the 14 cancer types. For each panel, the x-axis represents the predicted mutation rates and the y-axis denotes the actual mutation rates. **(A)** BLCA**; (B)** BRCA; **(C)** COAD; **(D)** ESCA; **(E)** HNSC; **(F)** KICH; **(G)** KIRC; **(H)** KIRP; **(I)** LIHC; **(J)** LUAD; **(K)** LUSC; **(L)** PRAD; **(M)** STAD; **(N)** THCA.

For each cancer type, a linear regression model is trained to predict the mutation rate using expression data in the same cancer sample, of selected genes from some EC subclasses as discussed above. The detailed objective function is described in the Methods section. To ensure the quality of each trained model, we have randomly selected 2/3 of the samples as the training data, and used the remaining 1/3 as the test data. Figure 2 shows the predicted values by the best trained model vs. the actual mutation rates across all samples for each cancer type. Similar plots for the test samples are given in Figure S1. Table 1 summarizes the level of contribution by genes of each EC subclass to the regression result for each cancer type, with the detailed information of the 14 models being given in Table S2.

**Table 1:** Estimated contribution by genes in each EC subclass to the linear model for each of the 14 cancer types.

**Cancer name**	**EC subclasses used**	**Contributions by each EC class**
BLCA	3.1.1	2.06E-22
	3.1.4	2.02E-26
	3.4.21	4.88E-13
	3.2.1	1.46E-21
BRCA	2.3.1	3.52E-21
	2.4.1	2.11E-24
	2.7.1	2.41E-14
	2.7.7	2.65E-18
COAD	2.7.1	1.18E-14
	2.4.1	5.97E-13
	3.1.3	2.44E-20
	3.4.21	3.02E-16
ESCA	2.1.1	4.55E-71
	2.3.1	7.00E-04
	2.4.1	5.07E-07
	2.6.1	9.62E-22
HNSC	2.7.1	2.94E-13
	2.4.1	7.11E-19
	3.1.3	5.58E-14
	3.4.21	2.51E-16
KICH	2.7.7	2.09E-05
	2.1.1	1.78E-16
	2.5.1	4.37E-05
	2.8.2	6.96E-06
KIRC	3.1.1	1.16E-10
	3.1.3	9.65E-17
	3.2.1	2.20E-10
	3.4.21	5.91E-28
KIRP	3.1.1	8.78E-14
	3.1.3	6.68E-07
	3.4.21	1.60E-23
	3.6.3	6.95E-16
LIHC	2.4.1	1.41E-21
	3.1.3	1.44E-12
	3.4.21	2.33E-12
	2.3.1	6.07E-25
LUAD	3.1.3	1.65E-63
	3.1.4	3.48E-07
	3.4.21	5.55E-18
	3.6.3	5.10E-35
LUSC	3.1.1	5.77E-43
	3.1.3	2.09E-24
	3.4.21	1.78E-26
	3.6.3	8.40E-22
PRAD	2.7.1	2.74E-41
	2.4.1	1.66E-11
	3.4.21	5.10E-14
	3.1.3	5.27E-05
STAD	2.7.1	5.47E-23
	2.4.1	1.34E-15
	3.1.3	5.06E-16
	3.4.21	8.40E-08
THCA	2.7.1	2.27E-31
	2.4.1	5.53E-12
	3.1.3	6.47E-11
	3.4.21	1.05E-06

### Prediction Validation

We have conducted the following analyses to provide supporting data to our prediction model.

#### Validation Against Fatty Acid Synthesis Genes

It is well-known that fatty acids serve as antioxidants in cancer and other disease cells (Richard et al., 2008; Freitas et al., 2017). Hence, we anticipate that our predictor should have some level of correlation with the fatty acid synthesis process. Table 2 shows the best correlation coefficient between our predictor and one of the four fatty acid synthesis genes (see Methods) across the 14 cancer types, with Table 3 giving the statistical significance of the observed correlations.

**Table 2:** Correlation coefficients between our oxidative-stress predictor and the fatty acid synthesis gene across 14 cancer types.

**Cancer name**	**Correlation coefficient**	**Gene name**
BLCA	0.77	FASN
BRCA	0.64	ACAT2
COAD	0.89	ACAT1
ESCA	0.99	ACAT2
HNSC	0.92	MCAT
KICH	0.98	ACAT2
KIRC	0.68	ACAT1
KIRP	0.8	ACAT1
LIHC	0.73	FASN
LUAD	0.81	ACAT2
LUSC	0.69	FASN
PRAD	0.72	FASN
STAD	0.66	MCAT
THCA	0.79	ACAT1

**Table 3:** Statistical significance for the observed correlation coefficient in Table 2 across 14 cancer types.

**Cancer name**	**ACAT1**	**MCAT**	**ACAT2**	**FASN**
BLCA	1.43E-01	1.44E-01	8.39E-02	4.36E-02
BRCA	2.60E-04	9.02E-02	7.97E-15	1.64E-02
COAD	1.10E-03	2.88E-06	8.34E-05	3.07E-01
ESCA	8.21E-01	8.18E-01	4.84E-01	8.06E-01
HNSC	4.30E-01	1.89E-09	2.43E-01	5.64E-02
KICH	1.29E-01	5.44E-01	8.24E-03	9.24E-01
KIRC	4.27E-03	3.35E-02	2.04E-01	1.42E-01
KIRP	1.55E-03	6.18E-02	7.80E-01	9.07E-02
LIHC	5.70E-02	5.22E-01	4.62E-01	2.81E-04
LUAD	9.87E-01	5.16E-03	5.63E-05	2.12E-05
LUSC	8.29E-01	4.06E-01	4.05E-02	3.48E-02
PRAD	3.38E-02	9.83E-01	9.33E-07	2.02E-01
STAD	8.52E-01	6.19E-04	8.79E-03	7.84E-01
THCA	2.87E-02	2.97E-01	1.60E-01	1.86E-01

#### Validation Against Mucin Genes

Mucins have been found to have elevated levels across numerous cancer types and are known to have anti-oxidation roles (Takeyama et al., 2000; Wu et al., 2009; Dewald et al., 2016). We have examined their expression levels and our predictor. Table 4 shows the best correlation coefficient between our predictor and one of the mucin genes across the 14 cancer types, with Table 5 giving the statistical significance of the observed correlations. And the statistical significance of the observed correlations for each Mucin genes is given in Table S3 in detail.

**Table 4:** Correlation coefficients between our oxidative-stress predictor and mucin genes across 14 cancer types.

**Cancer name**	**Correlation coefficient**	**Gene name**
BLCA	0.69	MUC15
BRCA	0.8	MUC20
COAD	0.75	MUC5B
ESCA	0.87	MUC17
HNSC	0.86	MUC3A
KICH	0.91	MUC12
KIRC	0.83	MUC16
KIRP	0.97	MUC12
LIHC	0.72	MUC20
LUAD	0.94	MUC22
LUSC	0.92	MUC4, MUC20
PRAD	0.76	MUC6
STAD	0.88	MUC7
THCA	0.88	MUC15

**Table 5:** Statistical significance for the observed correlation coefficients in Table 4 across 14 cancer types.

**Cancer name**	**Statistics significance**	**Gene name**
BLCA	2.40E-13	MUC15
BRCA	4.01E-11	MUC20
COAD	2.04E-12	MUC5B
ESCA	8.74E-07	MUC17
HNSC	8.74E-07	MUC3A
KICH	1.10E-04	MUC12
KIRC	3.95E-07	MUC6
KIRP	9.38E-06	MUC12
LIHC	2.96E-08	MUC1
LUAD	6.28E-07	MUC22
LUSC	8.48E-11	MUC1
PRAD	2.85E-04	MUC6
STAD	3.40E-07	MUC17
THCA	2.27E-03	MUC15

#### Validation Against Glutathione Synthesis Genes

Glutathione is a molecule human cells use as a key antioxidant. We have examined their expression levels and our predictor. Table 6 shows the R2 values between our predictor and the glutathione synthesis genes across the 14 cancer types, with Table 7 giving the statistical significance of the observed correlations.

**Table 6:** Correlation coefficients between our predictor and the glutathione synthesis genes across 14 cancer types.

**Cancer name**	**Correlation coefficient**	**Gene name**
BLCA	0.63	GCLM
BRCA	0.57	GCLM
COAD	0.94	GSS
ESCA	0.78	GCLC
HNSC	0.77	GCLM
KICH	0.45	GCLC
KIRC	0.74	GSS
KIRP	0.75	GSS
LIHC	0.66	GCLC
LUAD	0.74	GCLM
LUSC	0.75	GSS
PRAD	0.82	GCLC
STAD	0.79	GCLM
THCA	0.75	GCLC

**Table 7:** Statistical significance for observed correlation coefficient in Table 6 across 14 cancer types.

**Cancer name**	**GCLC**	**GSS**	**GCLM**
BLCA	3.56E-01	7.67E-03	1.12E-07
BRCA	3.07E-01	4.49E-01	5.16E-09
COAD	8.20E-01	1.53E-05	3.14E-02
ESCA	3.61E-14	4.09E-01	5.25E-01
HNSC	4.62E-01	3.05E-04	1.47E-06
KICH	1.38E-01	9.81E-01	7.89E-01
KIRC	5.90E-01	3.65E-05	3.67E-03
KIRP	7.88E-01	3.42E-07	7.88E-01
LIHC	5.50E-01	6.13E-06	9.30E-02
LUAD	1.88E-03	3.17E-02	6.50E-02
LUSC	4.62E-02	2.50E-29	3.15E-01
PRAD	1.11E-01	7.47E-02	4.29E-04
STAD	3.24E-04	1.74E-07	1.21E-08
THCA	9.80E-04	1.92E-01	2.08E-02

It is noteworthy that our predictor is designed to predict the level of oxidative stress; and each of the above three groups of genes is known to be associated with the level of oxidative stress and not involved in the training dataset. The observed strong correlations between our predicted oxidative stress levels and the expression levels of each such group, along with significant *p*-values, provide strong support for that our predictor captures the anti-oxidative level from different independent aspects, hence indicating the validity of our trained predictor as an indicator for the level of oxidative stress.

### Application

**Figure 3:**
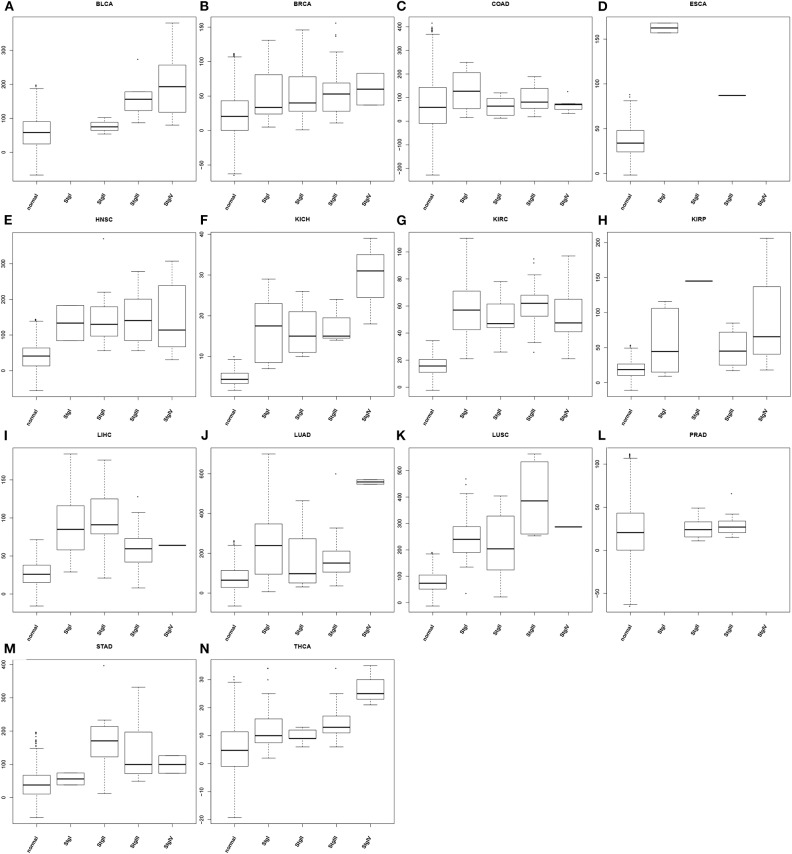
Boxplots of predicted oxidative stress levels across 4 cancer stages (when data are available) along with matching controls for each of the 14 cancer types. **(A)** BLCA**; (B)** BRCA; **(C)** COAD; **(D)** ESCA; **(E)** HNSC; **(F)** KICH; **(G)** KIRC; **(H)** KIRP; **(I)** LIHC; **(J)** LUAD; **(K)** LUSC; **(L)** PRAD; **(M)** STAD; **(N)** THCA.

To demonstrate the utility of our predictor, we have calculated the average oxidative stress levels for each of the four stages vs. the matching controls in each of the 14 cancer types, as detailed in Figure 3. From the figure, we can see: (1) cancer tissue cells have elevated oxidative-stress levels than matching controls in all of 14 cancer types; and (2) the oxidative stress level tends to progressively increase as a cancer advances from stage 1 through stage 4, in 10 out of 14 cancer types with at most one predicted level of oxidative stress being out of order. This is clearly consistent with our general understanding about cancer progression.

We have also conducted co-expression analyses to find genes that strongly correlate with our predictor in each cancer type and analyzed the pathways enriched by such genes. Table S4 gives the up-to top 100 enriched pathways with *p* < 0.05 in each cancer. We note that the enriched pathways are highly consistent across different cancer types. Furthermore, we note based on extensive literature search that a majority of the enriched pathways, marked in bold letters, is: (1) involved in anti-oxidation activities, (2) induced by oxidative stress, (3) induces oxidative stress, (4) involved in reactive oxidative stress signaling, and/or (5) is altered by oxidative stress. While these data provide supporting data to our predictor, pathways not known to be oxidative stress related may provide useful candidates for elucidation of the overall oxidative-stress responding mechanisms in cancer cells.

## Discussion

Quantitative assessment of the intracellular oxidative stress will prove to be an invaluable tool for elucidation of the possible causes of various changes in cancer cells, including extensive metabolic reprogramming. However, the problem proves to be very challenging because there are large numbers of contributors to both the total oxidizing power and the anti-oxidizing capacity in human cells. Previous studies tend to focus on oxidative stress induced by specific molecular species such as H2O_2_ or lipid radicals rather than the overall oxidative stress level. To the best our knowledge, our work is the first computational tool for estimating the oxidative stress level. The basic premise of our tool is that the genomic mutation level is strongly associated with the overall intracellular oxidative stress level. The second premise is that many enzymes have anti-oxidation capacity as reported by numerous authors (Sies, 1997; Rajput et al., 2013).

One surprising result in our regression analyses is that genes in two EC classes, EC 2.- (transferases) and EC 3.- (hydrolases), specifically four subclasses of these two, can be used to well interpret the mutation rate in each cancer sample by a linear combination of their expressions with high statistical significance. This strongly suggests that all the enzymes encoded by these genes play roles in cellular anti-oxidation, which is clearly a novel discovery and warrants further investigation. Furthermore, different cancer types tend to use a distinct combination of genes from four subclasses of enzymes strongly suggest that these cancer types may encounter different types of oxidative stress, hence using different combinations of anti-oxidation enzymes. This also warrants further study regarding why genes in different enzyme classes show strong correlations with mutation rates in different cancer types, therefore to understand the detailed mechanisms of their anti-oxidative functions possibly for different types of oxidants.

As mentioned in the Introduction, oxidative stress may arise from different molecular species such reactive oxygen species, reactive nitrogen species, reactive lipid species and various free radicals. Different molecular species might be utilized to consume specific types of oxidizing molecules. By showing that three independent classes of molecules all have statistically significant correlation coefficients with our general-purpose predictor for oxidative stress, we clearly have strong support for the validity of our predictor.

The availability of this new tool make open new doors for studying impact of oxidative stress on various chronic inflamed diseases, including cancer, including (i) elucidation of all metabolic processes, particularly reprogrammed metabolisms observed in cancer and other diseases, that are statistically associated with the level of oxidative stress, hence providing a new capability for detection of the possible causes of various altered metabolisms, and (ii) systematic analyses of different classes of enzymes in terms of their anti-oxidative roles, which could provide potentially powerful and new targets for treating various chronic diseases, including cancer.

We anticipate that our predictor will prove a powerful tool useful for elucidation of causes of variety of systematic changes, including metabolic reprogramming to gain in-depth understanding of why specific metabolic pathways are reprogrammed and certain cellular functions tend to be repressed or hyper-activated in cancer.

## Data and Methods

### Gene-Expression Data and Genomic Mutation Data

Gene expression and genomic mutation data of 14 cancer types: BLCA, BRCA, COAD, ESCA, HNSC, KICH, KIRC, KIRP, LIHC, LUAD, LUSC, PRAD, STAD, and THCA were downloaded from the TCGA data portal^1^. The detailed information of these data is summarized in Table 8.

**Table 8:** Information for 14 cancer types.

**Cancer name**	**Number of cancer samples**	**Number of control samples**
BLCA	408	20
BRCA	1,092	114
COAD	456	42
ESCA	164	12
HNSC	501	45
KICH	66	25
KIRC	530	73
KIRP	289	33
LICH	371	51
LUAD	515	60
LUSC	501	50
PRAD	495	53
STAD	380	33
THCA	502	59

Genomic mutation data were derived from the whole-exome sequencing data. Specifically, somatic changes are identified through comparing allele frequencies in the aligned DNA sequences of each cancer and the matching normal samples, using the GDC DNA-Seq analysis pipeline (GDC DNA-Seq analysis pipeline: https://docs.gdc.cancer.gov/Data/Bioinformatics_Pipelines/DNA_Seq_Variant_Calling_Pipeline). MuTect2 (GDC MuTect2: https://docs.gdc.cancer.gov/Data/Bioinformatics_Pipelines/DNA_Seq_Variant_Calling_Pipeline/#somatic-variant-calling-workflow) is used to call each mutation using the default parameters.

Human enzyme information, including gene names and the relevant enzyme classes, is downloaded from BRENDA (BRENDA links: https://www.brenda-enzymes.org/).

### Synthesis of Fatty Acids

We have used the four fatty-acid synthesis genes: *FASN* (Fatty Acid Synthase)*, ACAT1* (Acetyl-CoA Acetyltransferase 1)*, ACAT2* (Acetyl-CoA Acetyltransferase 2), and *MCAT* (Malonyl-CoA-Acyl Carrier Protein Transacylase) to reflect the level of fatty acid synthesis, and have calculated the Pearson correlation coefficient between the expressions of one of them and our predictor.

### Mucin Genes

Human has 20 mucin genes, namely *MUC1, MUC2, MUC8, MUC12, MUC13, MUC15, MUC16, MUC17, MUC19, MUC20, MUC21, MUC22, MUC3A, MUC3B, MUC4, MUC5AC, MUC5B, MUC6, MUC7, and MUCL1*. To assess their overall correlation with our predictor, we have conducted the Pearson correlation coefficient between the expression of each mucin gene and our oxidative stress predictor.

### Glutathione Synthesis

We have used the three biosynthesis genes: *GCLC* (glutamate-cysteine ligase catalytic subunit), *GCLM* (glutamate-cysteine ligase modifier subunit) and *GSS* (glutathione synthase) to reflect the level of glutathione synthesis. As above, we have calculated the Pearson correlation coefficient between the expressions of the glutathione synthesis genes and our oxidative stress predictor.

### Differential Expression Analyses

We have used edgeR in the R package (edgeR package: https://www.r-project.org/) to determine if a gene is differentially expressed in cancer vs. control samples of the same cancer type. *T*-test is applied to estimate the statistical significance of each gene considered as differentially expressed, using 0.05 as the cut-off.

### Linear Regression Analysis

We have conducted a linear regression analysis of the observed mutation rates, ***Y*_*n*_**, in n samples of a specified cancer type against the expressions of m selected genes across n samples, ***X*_*n, m*_**, so that residual ||ε|| is as small as possible as defined below:

$$\begin{array}{l} Y=XB+\;\varepsilon \end{array}$$

where ***B*_*m*_** is a coefficient vector with its m values to be determined through solving this optimization problem. To avoid using too many genes in the regression analysis, we have included a penalty for penalizing using more variables than necessary.

$$\begin{array}{l} Y=XB+\;\varepsilon +\lambda_{m} \end{array}$$

where λ is an (adjustable) constant. This problem can be solved using a least squared regression analyses in the following form:

$$\begin{array}{l} {\text{argmin}}_{B\epsilon R^{m}}{\left(Y-XB-\lambda_{m}\right)}^{2} \end{array}$$

For each ***Y***, we have retrieved the number of point mutations per cancer genome. Then for the n cancer genomes of each of the 14 cancer types, we have n numbers, namely the mutation number divided by the number of genes (20,000), which gives rise to the ***Y***. For the ith row of ***X***, we have retrieved the expression data of m selected genes in the ith sample of the same cancer type. For each regression analysis, we have used the R package to solve the minimization problem. The regression result has a *p*-value associated with each of the m values of ***B***. We then remove those genes with insignificant *p*-values, i.e., > 0.05, for the second round of regression analyses mentioned in the Results section.

### Correlation Analyses

We have used Pearson correlation coefficient to calculate the linear correlation between two lists of numbers, with one being the combined gene-expression data and the other being mutation rates of the same samples.

### Pathway Enrichment Analysis

We have conducted a pathway enrichment analysis over a given set of genes found to be strongly correlated with our predictor for oxidative stress using DAVID tool (David links: https://david.ncifcrf.gov/) against the combined database of GO/Biological Process, KEGG and Reactome pathways. A pathway is considered enriched if tits adjusted *p* < 0.05.

### Assessment of the Level of Contribution by Each Enzyme Subclass in Regression Model

For each derived regression model against selected enzymes, the following is used to estimate the level of contribution by each subclass of enzymes. The package used for linear repression construction provides a *p*-value for each selected gene, indicating the level of the gene's contribution to the regression result with smaller *p*-value representing higher level of contribution. We then use the product of the *p*-values of the selected genes from the same enzyme subclass as the *p*-value of the subclass.

## Software Availability

Our predictor is written in R language and is available upon request.

## Data Availability Statement

BLCA, BRCA, COAD, ESCA, HNSC, KICH, KIRC, KIRP, LIHC, LUAD, LUSC, PRAD, STAD, and THCA were downloaded from the TCGA data portal (TCGA data portal: https://portal.gdc.cancer.gov/). Human enzyme information, including gene names and the relevant enzyme classes, is downloaded from BRENDA (BRENDA links: https://www.brenda-enzymes.org/).

## Author Contributions

LL designed the method, carried out computational analyses, analyzed computational results, and wrote the article. HC supported in data analysis. YX conceived the project, reviewed computational analyses, and revised the article.

## Conflict of Interest

The authors declare that the research was conducted in the absence of any commercial or financial relationships that could be construed as a potential conflict of interest.

## Footnotes

^1^TCGA data portal: https://portal.gdc.cancer.gov/
