# Supplementary material for: Quantitative Estimation of Oxidative Stress in Cancer Tissue Cells Through Gene Expression Data Analyses
Source: Front Genet. 2020 May 19;11:494. doi: 10.3389/fgene.2020.00494 (PMC7263278; doi:10.3389/fgene.2020.00494)
Supplement: Supplementary file 1 [file Data_Sheet_1.pdf]

Supplementary Material

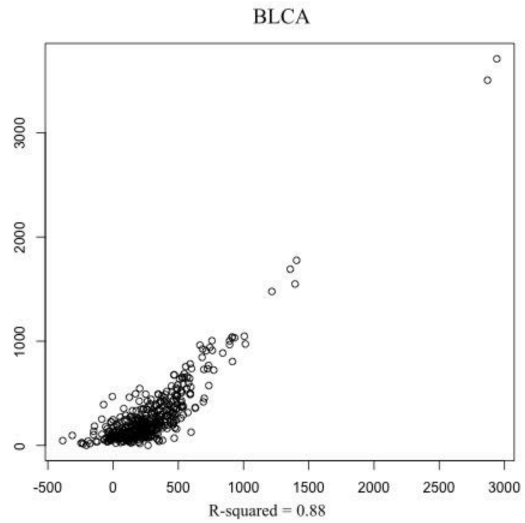

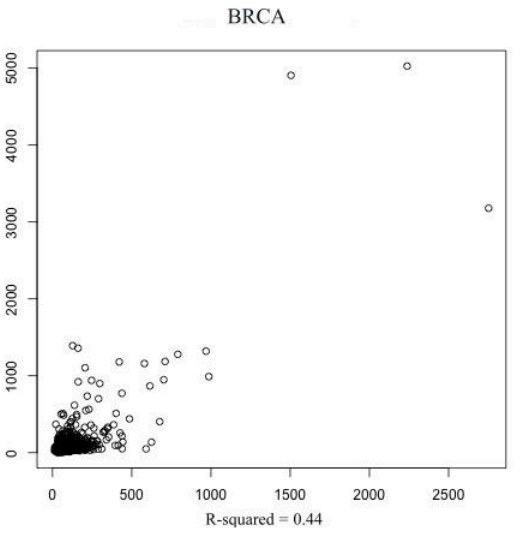

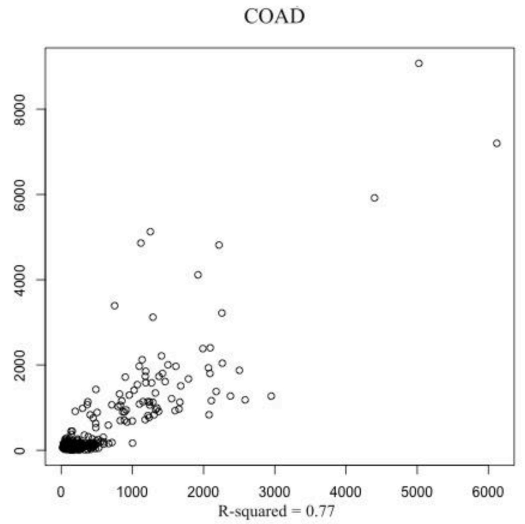

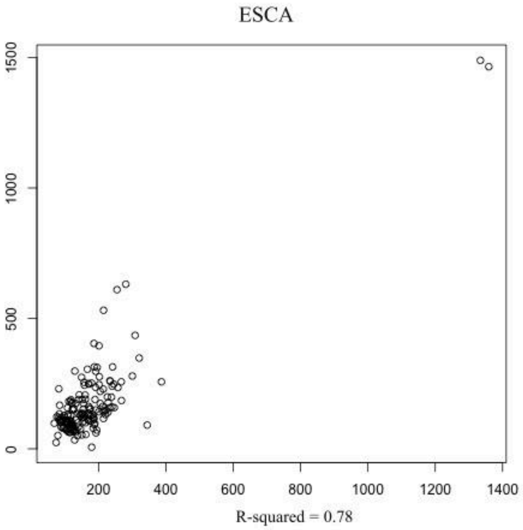

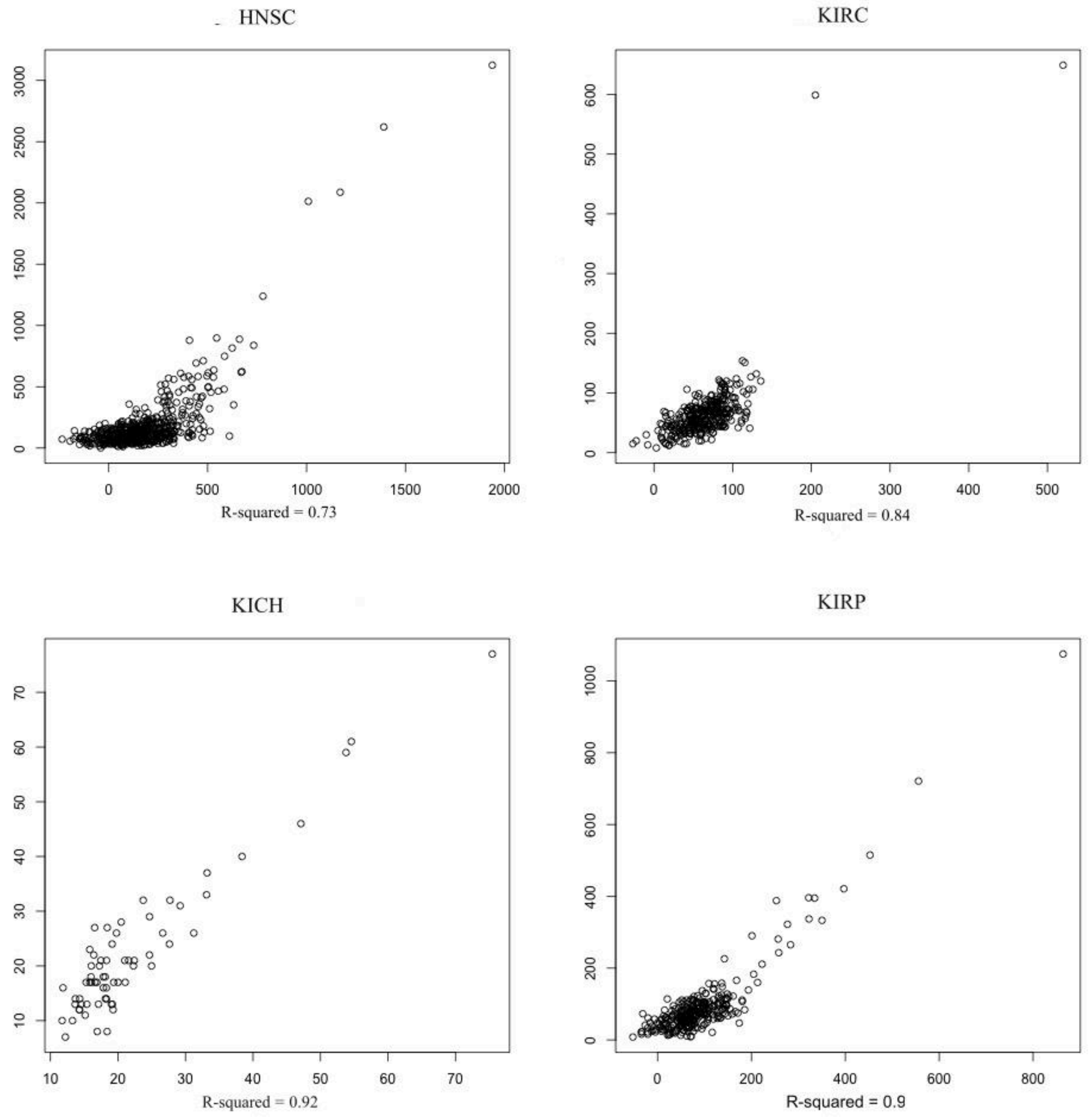
HNSC
R-squared = 0.73
KIRC
R-squared = 0.84
KICH
R-squared = 0.92
KIRP
R-squared = 0.9

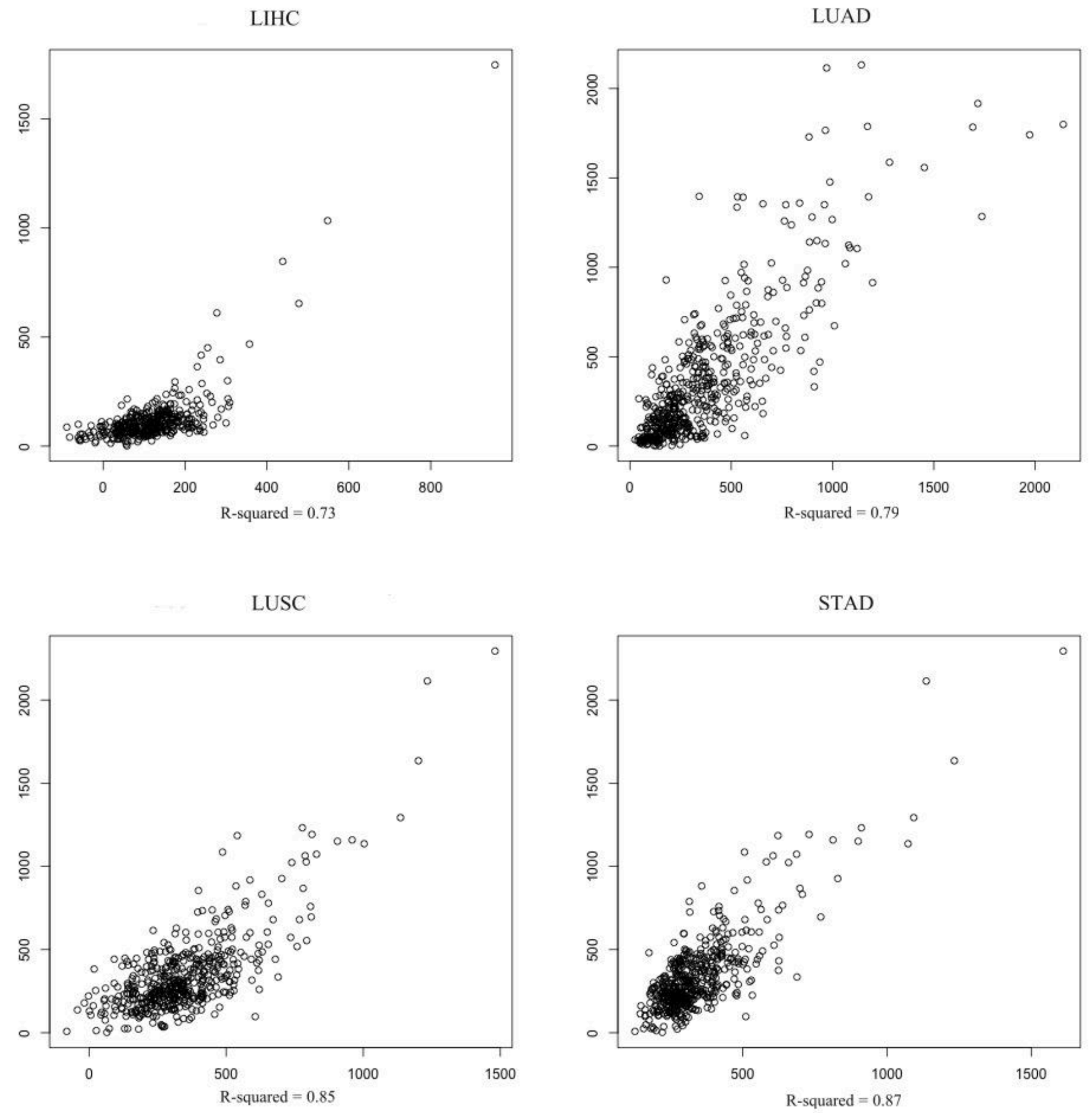

LIHC
R-squared = 0.73
LUAD
R-squared = 0.79
LUSC
R-squared = 0.85
STAD
R-squared = 0.87

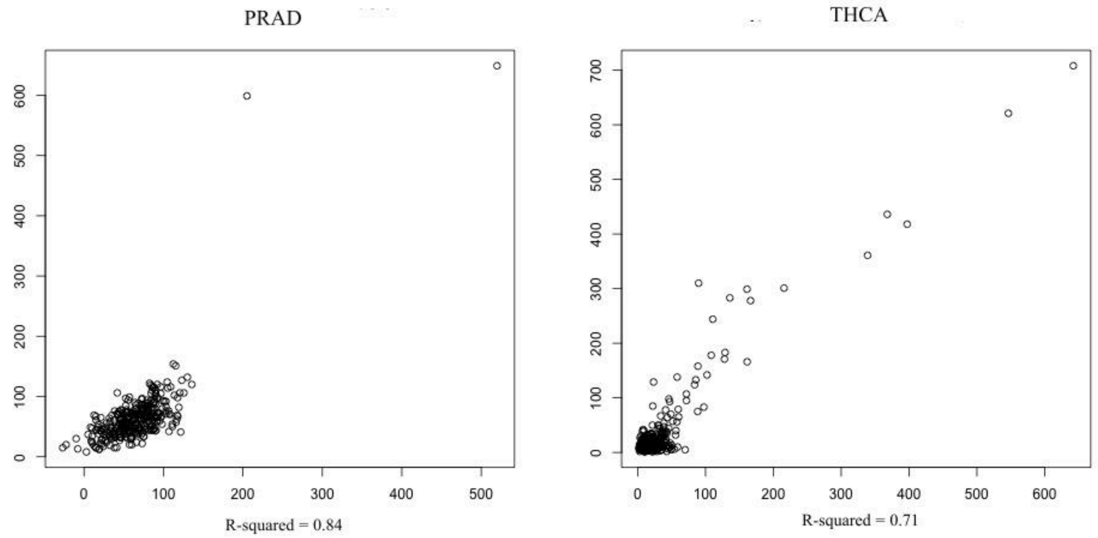

Figure S1: Scatter plots for predicted oxidative stress level vs. mutation rates in the test samples in 14 cancer types. For each panel, the x-axis represents the predicted oxidative stress level and the y-axis denotes the actual mutation rate.

Table S1: Genes selected from each regression model for each of the 14 cancer types, based on their level of contribution to the regression result as measured using p-values.

Cancer	Gene names
**BLCA**	ACP1, AGK, B3GALT2, B4GALT2, B4GALT3, B4GALT4, BPGM, C1R, C1S, C2, CDC14A, CDC14B, CELA3A, CFB, CFD, CFI, CHPF, CLP1, CMA1, CTRB1, CTSG, DCK, DUSP1, DUSP14, DUSP6, ELANE, ETNK1, ETNK2, EXT1, EXT2, F10, F11, F12, F2, F7F9, FUK, FUT8, GALK1, GALK2, GNE, GZMA, GZMB, HTRA2, IMPA1, IMPA2, INPP5A, INPP5B, INPPL1, KLK3, KLKB1, LHPP, MASP2, MBTPS1, NAGK, PGP, PHPT1, PLAT, PLAU, PLG, PLPP1, PLPP2, PLPP3, PPM1D, PPP1CA, PPP3CA, PPP3CB, PPP5C, PROC, PRSS1, PRSS2, PRSS3, PTPN5, PTPRJ, PTPRM, PTPRN,RELN, RNGTT, SGPP1, SGPP2, TIGAR, UGT8
**BRCA**	ACAA2, ADK, ADPGK, AGK, B3GNT2, B4GALT1, B4GALT2, B4GALT, B4GALT4, B4GALT7, B4GAT1, CHPF, CHSY1, CLP1, CMAS, CPT1B, DBT, DCK, DGKA, DGUOK, DLAT, DOLK, ETNK1, ETNK2, EXT1, EXT2, EXTL1, EXTL2, EXTL3, FPGT, FUT8, GALT, GCAT, GCK, GNE, HK1, HK3, IPPK, ITPK1, ITPKB, LIPT2, NAGS, PDXK, PFKM, PGM2L1, POLR3D, PSTK, RFK, TKFC
**COAD**	ACP1, ALPL, LPPL2, B3GNT2, B4GALT1, B4GALT7, B4GAT1, BPGM, BPNT1, C1R, C1S, C2, CELA2A, CELA2B, CELA3A, CFB, CFD, CFI, CMA1, CSGALNACT1, CSGALNACT2, CTRB1, CTRL, CTSG, DGKD, ELANE, ENOPH1, EXT1, EXT2, EXTL1, EXTL2, F12, F2, FUT8, FUT9, G6PC, G6PC2, GBE1, GZMA, GZMB, HTRA2, INPP1, ITPK1, KLK3, KLKB1, MASP1, MASP2, MBTPS1, MINPP1, NADK, NT5C, NT5C2, NT5E, PFKFB1, PFKFB2, PFKFB4, PHOSPHO1, PHOSPHO2, PHPT1, PLAT, PLAU, PLG, PROC, PTEN, PTPN11, PTPN5, PTPRJ, PTPRM, RELN, SGPP1, SGPP2, TIGAR
**ESCA**	ALG2, B3GALT1, B3GALT2, B3GNT4, B3GNT7, B4GALT1, B4GALT2, B4GALT3, B4GALT4, CHPF, CHSY3, COQ3, CPT1A, CPT1B, ELOVL2, EXTL1, EXTL2, EXTL3, FUT8, GAMT, GCNT1, GNMT, LIPT2, MGAT5B, MTR, NAT10, OXSM, PEMT, PRMT5, SCP2, TAT
**HNSC**	A4GALT, ABO, ACP1, ADK, ADPGK, B3GALT2, B4GALT2, B4GALT3, B4GALT4, BPGM, C2, CELA2B, CELA3A, CERK, CFB, CMA1, COASY, CSGALNACT1, CSGALNACT2, CTRB1, CTRL, CTSG, DGKA, DGUOK, DOLK, ELANE, F10, F2, FBP1, FBP2, FUK, GCK, GK, GK2, GLYCTK, GNE, GZMA, GZMB, HKDC1, HTRA2, IMPA1, IMPA2, IPMK, IPPK, KHK, KLK3, KLKB1, LHPP, MASP1, MASP2, MBTPS1, MVK, NADK, NAGK, NMRK1, NT5C2, NT5E, OCRL, PDXP, PFKL, PFKM, PFKP, PGM2L1, PGP, PHPT1, PIK3C2A, PIK3C2B, PIK3C3, PIK3CA, PIK3CD, PIKFYVE, PIP4K2A, PIP4K2C, PKM, PLAT, PLAU, PPM1D, PPP5C, PROC, PSPH, PSTK, PTPN1, PTPN5, PTPN7, RBKS, RELN, RFK, SGPP1, SGPP2, SYNJ1, SYNJ2, TIGAR, TK1, TK2, TKFC, UGT8, XYLB
**KICH**	CHST1, CMAS, COQ3, DNMT1, DNMT3A, DNMT3B, FDPS, FLAD1, FPGT, SMS, SRM, UAP1, UAP1L1

**KIRC**	ACHE, ACP1, ACPP, AGL, BPGM, BPNT1, CEL, CES2, CES5A, CFD, CFI, CHI3L1, CHIA, CMA1, CMBL, CTSG, DUSP10, ENGASE, FBP1, FBP2, FUCA1, FUCA2, GAA, GALC, GANAB, GBA, GBA3, GLA, GZMA, GZMB, H6PD, HPSE, HPSE2, HYAL1, IDUA, IMPA1, IMPA2, INPP1, INPP4A, INPP4B, INPP5B, KLK3, LCT, LHPP, LIPA, LIPE, MAN2A2, MAN2B1, MANBA, MASP1, MBTPS1, MGAM, MGLL, MOGS, NAGA, NAGLU, NANP, NCEH1, NEU1, NEU3, NT5M, PAFAH2, PDXP, PGLS, PGP, PHPT1, PLA2G4B, PLA2G4E, PLAT, PLAU, PLB1, PLG, PNLIP, PPM1D, PPP5C, PROC, PSPH, PTEN, PTPN1, PTPN5, PTPN6, PTPRB, RELN, SGPP1, SGPP2, SI, SPAM1, SYNJ1, SYNJ2, TIGAR, TREH
**KIRP**	CES1, CES2m, CES5A, CEL, LIPA, MGLL, LIPF, LIPG, PNLIP, PNLIPRP1, PNLIPRP2, H6PD, PGLS, LPL, PLA2G1B, PLA2G3, PLA2G4B, CMBL, PAFAH1B2, PAFAH1B3, LYPLA1, LYPLA2, PLB1, ACHE, PHPT1, LHPP, SGPP1, SGPP2, ALPI, ALPPL2, FBP1, FBP2, BPGM, PPM1A, ACP2, ACP5, PSPH, RNGTT, OCRL, INPP5E, TIGAR, ACP1, CTRL, RELN, CTRB1, MASP2, HTRA2, KLKB1, ELANE, F12, PRSS1, PRSS2, C1S, CFI, CFD, CFB, F2, F10, PLAT, PLG, CELA3B, PLAU, KLK3, ATP4B, ATP5A1, ATP5O, ATP6V0A2, ATP6V0D2, DHX58, TCIRG1, CFTR, ATP2B2, ATP2B4, ATP1A1, ATP1A2
**LIHC**	TA, SLC33A1, ELOVL2, NAT10, LPCAT4, NAGS, HADHB, ACAA2, DBT, FUT4, FUT7, B4GALT1, OGT, B4GALT4, B4GALT3, B4GALT2, B3GALT2, B3GALT1, CSGALNACT2, CSGALNACT1, EXTL2, EXTL1, EXTL3, CHSY1, CHPF2, ABO, PLOD3, FUT8, SGPP1, SGPP2, FBP1, FBP2, BPGM, PPP5C, PGP, PLPP1, PLPP2, PLPP3, TIGAR, ACP1, PTPRF, PTPRJ, DUSP16, PTPN5, NT5E, NT5M, NT5C1, INPP5J, INPP1, PTEN, BPNT1, PDXP, PHOSPHO2, PHOSPHO1, ENOPH1,MBTPS1, CTSG, F7, CMA1, C1S, CFB, PROC, CELA3A, PLAU, KLK3
**LUAD**	PGP, IMPA, PSPH, RNGTT, INPP5B, INPP5E, TIGAR, DUSP16, PTPN5, ENPP6, PLCB2, PLCB3, SMPD, SMPD3, PDE1A, PDE1C, PDE1B, PDE7B, PDE9A, ENPP2, PLD2, NAGPA, CTRL, MASP1, RELN, CTRB1, MASP2, HTRA2, MBTPS1, CTSG, F9, KLKB1, ELANE, F12, PRSS1, PRSS2, C1R, C1S, C2, CFI, CFD, CFB, F2, F10, PLAT, PROC, PLG, CELA2B, CELA2A, PLAU, KLK3, GZMB, ATP4A, ATP4B, ATP5B, ATP5C1, ATP5D, ATP5E, ATP5F, ATP5G2, ATP5I, ATP5J, ATP6V1B1, ATP6V1C1, ATP6V1E1, ATP6AP1, ATP5O, MT-ATP8, ATP6V1F, TCIRG1, ATP5H, DDX58, ATP6V1H, DHX58, ATP6V1G3, CFTR, ATP2A1, ATP2A2, ATP2B1, ATP2B4, ATP1A1, ATP1A3
**LUSC**	PGLS, LPL, CMBL, LYPLA1, LYPLA2, PLA2G15, PLB1, ACHE, LIPE, PHPT1,LHPP, SGPP1, SGPP2, ALPPL2, BPGM, PPM1B, PPP2CA, PPP3CB, ACP2, ACP4, NANP, PSPH, RNGTT, INPP5B, TIGAR, ACP1, CDC25B, PTPN6, PTPRR, NT5M, INPP1, PTEN, BPNT1, RELN, MASP2, HTRA2, MBTPS1, F9, KLKB1, ELANE, F12, C1R, C2, CFB, F2, PLAT, PROC, PLG, ATP4B, ATP5A1, ATP5B, ATP5, ATP6V0A1, ATP6AP1, MT-ATP6, MT-ATP8, TCIRG1, ATP5, ATP5L, CFTR, ATP2A1, ATP2B3, ATP1A1
**PRAD**	B4GALT1, OGT, B4GALT4, B4GALT3, B4GALT2, B3GALT2, EXTL1, EXTL3, EXT1, EXT2, CHPF, UGT8, FUT5, FUT8, B3GALNT1, DPM1, GBGT1, HKDC1, PGM2L1, DGKH, DOLK, PFKM, ITPKA, ITPKB, CER, IPPK, PSTK, XYLB, GCK, PHPT1, LHPP, SGPP1, SGPP2, ALPI, FBP1, FBP2, BPG, PPP5, PPM1D, PGP, ACPP, ACP6, NANP, PSPH, RNGTT, OCRL, SYNJ1, SYNJ2, INPP5, TIGA, ACP1, PTPN1, PTPN, CTRL, MASP1, REL, CTRB1, MASP2, HTRA2, MBTPS1, CTSG, F7, F9, F11, KLKB1, ELANE, F1, CMA1, PRSS1, C1R, C1S, C2, CFI, CFD, CFB, F2, F10, PLAT, PROC, PLG, CELA3A, CELA3, CELA2B, CELA2A, PLAU, KLK3, GZMA, GZMB
**STAD**	ABO, ACP, ACP2, ACPP, ADK, ADPGK, BPGM, C1R, C1, CERK, CHPF2, CHSY, CMA1, COASY, CTSG, DGKA, DGKB, DGKH, DGKI, DGUOK, DOLK, DPM1, DUSP1, ELANE, EXT1, EXT2, EXTL1, EXTL3, FBP1, F2, GBGT1, GCK, GK, GK2, GLYCTK, HK, HKDC1, IMPA1, IMPA, INPP5B, INPP5E, IPMK, IPPK, ITPK1, KHK, KL, LHPP, MVK, NADK, NANP, NMRK1, NT5C2, NT5E, NT5M, OCRL, PAPSS1, PAPSS2, PFKL, PGM2L1, PGP, PHPT1, PIK3C3, PIKFYVE, PIP4K2C, PKM, PPM1D, PRSS1, PRSS2, PRSS3, PSPH, PSTK, PTPN5, PTPRJ, PTPRN, RBKS, RNGTT, SGPP1, SGPP2, SYNJ2, TIGAR, TK1, TK2, TKFC, XYLB
**THCA**	ACP1, AGK, B3GALT2, B4GALT2, B4GALT3, B4GALT4, BPGM, C1R, C1S, C2, CDC14A, CDC14B, CELA3A, CFB, CFD, CFI, CHPF, CLP1, CMA1, CTRB1, CTSG, DCK, DUSP1, DUSP14, DUSP6, ELANE, ETNK1, ETNK2, EXT1, EXT2, F10, F11, F12, F2, F7, F9, FUK, FUT8, GALK1, GALK2, GNE, GZMA, GZMB, HTRA2, IMPA1, IMPA2, INPP5A, INPP5B, INPPL1, KLK3, KLKB1, LHPP, MASP2, MBTPS1, NAGK, PGP, PHPT1, PLAT, PLAU, PLG, PLPP1, PLPP2, PLPP3, PPM1D, PPP1CA, PPP3CA, PPP3CB, PPP5C, PROC, PRSS1, PRSS2, PRSS3, PTPN5, PTPRJ, PTPRM, PTPRN, RELN, RNGTT, SGPP1, SGPP2, TIGAR, UGT8

Table S2: Enzymes used in our linear regression analysis with gene number in each enzyme class.

Cancer	EC classes used	Number of genes used
**BLCA**	3.1.1	20

	3.1.4	12
	3.4.21	32
	3.2.1	17
**BRCA**	2.3.1	7
	2.4.1	15
	2.7.1	24
	2.7.7	4
**COAD**	2.7.1	3
	2.4.1	13
	3.1.3	27
	3.4.21	29
**ESCA**	2.1.1	6
	2.3.1	7
	2.4.1	17
	2.6.1	1
**HNSC**	2.7.1	41
	2.4.1	9
	3.1.3	24
	3.4.21	23
**KICH**	2.7.7	5
	2.1.1	4
	2.5.1	3
	2.8.2	1
**KIRC**	3.1.1	16
	3.1.3	32
	3.2.1	29
	3.4.21	14
**KIRP**	3.1.1	24
	3.1.3	18
	3.4.21	21
	3.6.3	12
**LIHC**	2.4.1	19
	3.1.3	27
	3.4.21	10

	2.3.1	9
**LUAD**	3.1.3	10
	3.1.4	13
	3.4.21	30
	3.6.3	30
**LUSC**	3.1.1	9
	3.1.3	24
	3.4.21	15
	3.6.3	15
**PRAD**	2.7.1	12
	2.4.1	18
	3.4.21	35
	3.1.3	24
**STAD**	2.7.1	37
	2.4.1	9
	3.1.3	29
	3.4.21	9
**THCA**	2.7.1	10
	2.4.1	9
	3.1.3	31
	3.4.21	32

Table S3: The statistical significance for correlations between our predictor and the mucin gene across 14 cancer types.

Cancer	MUC12	MUCL1	MUC5B	MUC1	MUC5AC	MUC22	MUC15	MUC21	MUC16	MUC17	MUC13	MUC7	MUC3A	MUC20	MUC6
**BLCA**	3.19E-01	1.65E-02	8.45E-01	2.40E-13	1.00E+00	4.96E-01	1.50E-02	2.47E-01	2.29E-01	6.18E-01	1.83E-01	1.42E-01	3.22E-02	1.59E-03	2.45E-01
**BRCA**	5.03E-01	3.00E-01	2.54E-02	9.52E-07	3.21E-01	1.81E-03	6.60E-03	8.24E-01	6.92E-01	1.46E-01	8.54E-05	1.94E-05	4.01E-11	7.14E-06	2.45E-01
**COAD**	2.39E-08	4.19E-02	5.05E-01	1.91E-02	2.15E-02	6.47E-01	4.90E-01	9.89E-02	4.95E-02	1.98E-01	2.04E-12	9.11E-01	1.11E-01	2.13E-08	1.37E-03
**ESCA**	1.51E-01	4.89E-01	4.09E-01	8.74E-07	1.51E-01	7.43E-01	4.48E-02	3.78E-02	1.02E-01	3.33E-01	5.20E-01	3.24E-01	9.23E-01	3.10E-01	7.37E-01
**HNSC**	1.19E-01	6.66E-01	2.80E-04	1.55E-22	2.29E-01	3.01E-02	3.90E-03	1.93E-04	3.02E-03	3.50E-03	3.57E-01	7.95E-02	2.30E-01	3.36E-01	8.72E-01
**KICH**	4.45E-02	8.65E-01	6.26E-01	1.10E-04	5.01E-01	9.99E-01	6.86E-01	3.44E-01	3.86E-01	2.97E-01	3.40E-01	4.15E-01	6.18E-01	2.51E-01	8.61E-02
**KIRC**	6.89E-02	8.91E-01	3.00E-01	4.50E-04	2.61E-01	4.25E-01	4.05E-04	1.94E-01	5.43E-01	2.54E-02	5.99E-05	9.64E-01	3.95E-07	5.66E-02	1.56E-01
**KIRP**	1.66E-01	1.76E-01	6.91E-01	5.88E-01	1.89E-01	7.73E-01	4.02E-01	8.91E-01	5.80E-01	8.19E-02	2.49E-02	8.06E-01	9.38E-06	5.55E-03	2.60E-01
**LIHC**	7.24E-01	3.52E-02	6.47E-01	2.96E-08	1.52E-01	7.84E-01	2.39E-04	6.97E-02	4.16E-01	6.59E-01	3.92E-03	1.22E-01	9.79E-01	2.19E-06	7.45E-03
**LUAD**	1.40E-01	5.59E-04	1.22E-03	6.28E-07	3.49E-01	9.22E-05	5.81E-01	1.33E-05	1.76E-02	7.61E-02	2.01E-02	1.16E-01	5.62E-01	1.62E-02	6.26E-02
**LUSC**	1.76E-02	5.98E-01	7.84E-02	8.48E-11	7.91E-01	1.72E-01	2.78E-05	4.12E-04	7.65E-03	5.24E-01	8.66E-01	1.17E-01	3.31E-03	2.58E-02	2.02E-01
**PRAD**	3.04E-01	1.27E-01	3.89E-01	9.33E-04	3.05E-01	1.04E-01	3.87E-02	1.08E-01	6.00E-02	2.23E-02	1.12E-03	1.41E-01	8.91E-03	7.21E-04	6.09E-02
**STAD**	6.48E-01	2.62E-02	2.01E-01	8.31E-04	1.09E-01	1.17E-02	5.35E-05	4.11E-02	4.84E-01	2.75E-01	3.40E-07	4.57E-01	4.64E-01	1.76E-02	4.99E-01
**THCA**	5.25E-01	4.27E-01	3.94E-01	2.27E-03	3.36E-01	7.42E-01	2.00E-01	5.73E-02	4.95E-01	1.54E-01	3.38E-01	5.71E-01	2.56E-01	3.42E-01	8.93E-01

Table S4: Pathways enriched for each of the 14 cancer types (p-value cutoff is 0.05), where pathway names in bold denote those known to be related to oxidative stress.

Database	Enriched pathways in BLCA	P-Value
KEGG_PATHWAY	**hsa04610:Complement and coagulation cascades**	2.38E-19
REACTOME_PATHWAY	**Activation of Matrix Metalloproteinases**	2.86E-09
REACTOME_PATHWAY	**Intrinsic Pathway of Fibrin Clot Formation**	7.99E-09
REACTOME_PATHWAY	Transport of gamma-carboxylated protein precursors from the endoplasmic reticulum to the Golgi apparatus	3.13E-07
REACTOME_PATHWAY	Gamma-carboxylation of protein precursors	5.19E-07
REACTOME_PATHWAY	Removal of aminoterminal propeptides from gamma-carboxylated proteins	5.19E-07
KEGG_PATHWAY	hsa05150:Staphylococcus aureus infection	1.62E-06
BIOCARTA	**h_intrinsicPathway:Intrinsic Prothrombin Activation Pathway**	1.90E-06
BIOCARTA	**h_plateletAppPathway:Platelet Amyloid Precursor Protein Pathway**	4.01E-06
BIOCARTA	**h_compPathway:Complement Pathway**	1.94E-05
BIOCARTA	**h_amiPathway:Acute Myocardial Infarction**	2.51E-05
REACTOME_PATHWAY	**Synthesis of IP2, IP, and Ins in the cytosol**	5.82E-05
KEGG_PATHWAY	**hsa00600:Sphingolipid metabolism**	1.45E-04
REACTOME_PATHWAY	**Sphingolipid de novo biosynthesis**	2.11E-04
REACTOME_PATHWAY	Regulation of Insulin-like Growth Factor (IGF) transport and uptake by Insulin-like Growth Factor Binding Proteins (IGFBPs)	4.45E-04
REACTOME_PATHWAY	**Extrinsic Pathway of Fibrin Clot Formation**	5.14E-04
KEGG_PATHWAY	**hsa00533:Glycosaminoglycan biosynthesis - keratan sulfate**	5.16E-04
BIOCARTA	**h_extrinsicPathway:Extrinsic Prothrombin Activation Pathway**	1.43E-03
BIOCARTA	**h_fibrinolysisPathway:Fibrinolysis Pathway**	1.43E-03
KEGG_PATHWAY	**hsa00601:Glycosphingolipid biosynthesis - lacto and neolacto series**	2.70E-03
REACTOME_PATHWAY	**Dissolution of Fibrin Clot**	3.86E-03
KEGG_PATHWAY	hsa01100:Metabolic pathways	4.19E-03
REACTOME_PATHWAY	**N-Glycan antennae elongation**	5.15E-03
KEGG_PATHWAY	**hsa00562:Inositol phosphate metabolism**	7.30E-03
REACTOME_PATHWAY	Cobalamin (Cbl, vitamin B12) transport and metabolism	1.00E-02
REACTOME_PATHWAY	**Common Pathway of Fibrin Clot Formation**	1.10E-02
KEGG_PATHWAY	**hsa00565:Ether lipid metabolism**	1.27E-02
REACTOME_PATHWAY	DARPP-32 events	1.51E-02
KEGG_PATHWAY	**hsa00520:Amino sugar and nucleotide sugar metabolism**	1.52E-02
REACTOME_PATHWAY	**Initial triggering of complement**	1.71E-02
REACTOME_PATHWAY	**Keratan sulfate biosynthesis**	1.74E-02
REACTOME_PATHWAY	**Regulation of Complement cascade**	1.74E-02
KEGG_PATHWAY	**hsa00564:Glycerophospholipid metabolism**	1.97E-02
KEGG_PATHWAY	**hsa04070:Phosphatidylinositol signaling system**	2.18E-02
BIOCARTA	**h_classicPathway:Classical Complement Pathway**	2.98E-02
REACTOME_PATHWAY	Negative regulation of MAPK pathway	3.10E-02
REACTOME_PATHWAY	**Alternative complement activation**	3.58E-02

Database	Enriched pathways in BRCA	P-Value
GOTERM_BP_DIRECT	**GO:0016311~dephosphorylation**	1.01E-22
GOTERM_BP_DIRECT	**GO:0006508~proteolysis**	1.27E-19
KEGG_PATHWAY	**hsa04610:Complement and coagulation cascades**	1.64E-15
GOTERM_BP_DIRECT	**GO:0042730~fibrinolysis**	2.10E-08
KEGG_PATHWAY	hsa05150:Staphylococcus aureus infection	3.85E-08
GOTERM_BP_DIRECT	**GO:0006003~fructose 2,6-bisphosphate metabolic process**	6.90E-07
REACTOME_PATHWAY	**Activation of Matrix Metalloproteinases**	1.29E-06
GOTERM_BP_DIRECT	**GO:0006956~complement activation**	1.63E-06
KEGG_PATHWAY	hsa01100:Metabolic pathways	3.25E-06
GOTERM_BP_DIRECT	**GO:0022617~extracellular matrix disassembly**	1.60E-05
GOTERM_BP_DIRECT	**GO:0006024~glycosaminoglycan biosynthetic process**	2.75E-05
GOTERM_BP_DIRECT	**GO:0015012~heparan sulfate proteoglycan biosynthetic process**	4.52E-05
GOTERM_BP_DIRECT	G**O:0035335~peptidyl-tyrosine dephosphorylation**	5.75E-05
KEGG_PATHWAY	**hsa00534:Glycosaminoglycan biosynthesis - heparan sulfate / heparin**	7.54E-05
GOTERM_BP_DIRECT	**GO:0015014~heparan sulfate proteoglycan biosynthetic process, polysaccharide chain biosynthetic process**	1.02E-04
GOTERM_BP_DIRECT	**GO:0051919~positive regulation of fibrinolysis**	1.02E-04
GOTERM_BP_DIRECT	**GO:0048008~platelet-derived growth factor receptor signaling pathway**	2.35E-04
REACTOME_PATHWAY	Regulation of Insulin-like Growth Factor (IGF) transport and uptake by Insulin-like Growth Factor Binding Proteins (IGFBPs)	2.46E-04
REACTOME_PATHWAY	**Intrinsic Pathway of Fibrin Clot Formation**	2.83E-04
KEGG_PATHWAY	**hsa00533:Glycosaminoglycan biosynthesis - keratan sulfate**	3.70E-04
GOTERM_BP_DIRECT	**GO:0006487~protein N-linked glycosylation**	5.69E-04
GOTERM_BP_DIRECT	**GO:0006000~fructose metabolic process**	7.54E-04
GOTERM_BP_DIRECT	**GO:0006958~complement activation, classical pathway**	7.74E-04
GOTERM_BP_DIRECT	**GO:0006094~gluconeogenesis**	8.13E-04
GOTERM_BP_DIRECT	**GO:0043647~inositol phosphate metabolic process**	9.86E-04
REACTOME_PATHWAY	**Initial triggering of complement**	9.96E-04
GOTERM_BP_DIRECT	**GO:0007596~blood coagulation**	1.02E-03
GOTERM_BP_DIRECT	**GO:0006486~protein glycosylation**	1.27E-03
GOTERM_BP_DIRECT	GO:0006195~purine nucleotide catabolic process	1.30E-03
REACTOME_PATHWAY	Purine catabolism	1.85E-03
KEGG_PATHWAY	hsa00**601:Glycosphingolipid biosynthesis - lacto and neolacto series**	1.96E-03
GOTERM_BP_DIRECT	**GO:0007597~blood coagulation, intrinsic pathway**	2.51E-03
REACTOME_PATHWAY	**Dissolution of Fibrin Clot**	2.60E-03
KEGG_PATHWAY	**hsa00760:Nicotinate and nicotinamide metabolism**	2.69E-03
GOTERM_BP_DIRECT	GO:0016485~protein processing	3.37E-03
KEGG_PATHWAY	**hsa00051:Fructose and mannose metabolism**	3.58E-03
GOTERM_BP_DIRECT	**GO:0046835~carbohydrate phosphorylation**	4.10E-03
GOTERM_BP_DIRECT	**GO:0001958~endochondral ossification**	5.22E-03
GOTERM_BP_DIRECT	GO:0061621~canonical glycolysis	5.22E-03

GOTERM_BP_DIRECT	**GO:0018146~keratan sulfate biosynthetic process**	6.04E-03
GOTERM_BP_DIRECT	**GO:0030449~regulation of complement activation**	6.91E-03
GOTERM_BP_DIRECT	**GO:0050653~chondroitin sulfate proteoglycan biosynthetic process, polysaccharide chain biosynthetic process**	8.32E-03
GOTERM_BP_DIRECT	**GO:0033692~cellular polysaccharide biosynthetic process**	8.32E-03
GOTERM_BP_DIRECT	**GO:0002542~Factor XII activation**	8.32E-03
GOTERM_BP_DIRECT	**GO:0006096~glycolytic process**	8.82E-03
REACTOME_PATHWAY	**Regulation of Complement cascade**	1.19E-02
REACTOME_PATHWAY	**Keratan sulfate biosynthesis**	1.19E-02
GOTERM_BP_DIRECT	**GO:0050651~dermatan sulfate proteoglycan biosynthetic process**	1.25E-02
GOTERM_BP_DIRECT	**GO:0019276~UDP-N-acetylgalactosamine metabolic process**	1.25E-02
GOTERM_BP_DIRECT	GO:0044267~cellular protein metabolic process	1.32E-02
GOTERM_BP_DIRECT	GO:0050900~leukocyte migration	1.44E-02
REACTOME_PATHWAY	Glycolysis	1.54E-02
GOTERM_BP_DIRECT	**GO:0006470~protein dephosphorylation**	1.57E-02
KEGG_PATHWAY	**hsa00532:Glycosaminoglycan biosynthesis - chondroitin sulfate / dermatan sulfate**	1.59E-02
GOTERM_BP_DIRECT	**GO:0006670~sphingosine metabolic process**	1.66E-02
GOTERM_BP_DIRECT	**GO:0033133~positive regulation of glucokinase activity**	2.07E-02
GOTERM_BP_DIRECT	**GO:0030311~poly-N-acetyllactosamine biosynthetic process**	2.07E-02
GOTERM_BP_DIRECT	**GO:0043456~regulation of pentose-phosphate shunt**	2.07E-02
KEGG_PATHWAY	**hsa00010:Glycolysis / Gluconeogenesis**	2.72E-02
GOTERM_BP_DIRECT	GO:0007586~digestion	2.84E-02
GOTERM_BP_DIRECT	**GO:0007411~axon guidance**	2.87E-02
GOTERM_BP_DIRECT	**GO:0001867~complement activation, lectin pathway**	2.88E-02
REACTOME_PATHWAY	Ficolins bind to repetitive carbohydrate structures on the target cell surface	2.94E-02
REACTOME_PATHWAY	**Alternative complement activation**	2.94E-02
KEGG_PATHWAY	**hsa04152:AMPK signaling pathway**	3.14E-02
KEGG_PATHWAY	**hsa00562:Inositol phosphate metabolism**	3.15E-02
GOTERM_BP_DIRECT	**GO:0014909~smooth muscle cell migration**	3.29E-02
GOTERM_BP_DIRECT	**GO:0050650~chondroitin sulfate proteoglycan biosynthetic process**	3.29E-02
KEGG_PATHWAY	**hsa00052:Galactose metabolis**m	3.41E-02
REACTOME_PATHWAY	**Lectin pathway of complement activation**	3.52E-02
GOTERM_BP_DIRECT	**GO:0005975~carbohydrate metabolic process**	3.61E-02
GOTERM_BP_DIRECT	**GO:0031639~plasminogen activation**	3.69E-02
GOTERM_BP_DIRECT	**GO:0031638~zymogen activation**	3.69E-02
GOTERM_BP_DIRECT	GO:0050778~positive regulation of immune response	3.69E-02
KEGG_PATHWAY	**hsa00500:Starch and sucrose metabolism**	4.06E-02
GOTERM_BP_DIRECT	**GO:0042355~L-fucose catabolic process**	4.09E-02
GOTERM_BP_DIRECT	**GO:0051156~glucose 6-phosphate metabolic process**	4.09E-02
GOTERM_BP_DIRECT	**GO:0051918~negative regulation of fibrinolysis**	4.09E-02
KEGG_PATHWAY	hsa05322:Systemic lupus erythematosus	4.11E-02
GOTERM_BP_DIRECT	GO:0050728~negative regulation of inflammatory response	4.30E-02

GOTERM_BP_DIRECT	**GO:0001503~ossification**	4.39E-02
GOTERM_BP_DIRECT	GO:0002003~angiotensin maturation	4.49E-02
GOTERM_BP_DIRECT	**GO:0009312~oligosaccharide biosynthetic process**	4.49E-02
GOTERM_BP_DIRECT	GO:0046135~pyrimidine nucleoside catabolic process	4.49E-02
GOTERM_BP_DIRECT	**GO:0046855~inositol phosphate dephosphorylation**	4.49E-02
GOTERM_BP_DIRECT	**GO:0017187~peptidyl-glutamic acid carboxylation**	4.49E-02
GOTERM_BP_DIRECT	GO:0033628~regulation of cell adhesion mediated by integrin	4.49E-02
REACTOME_PATHWAY	**Activation of C3 and C5**	4.66E-02
GOTERM_BP_DIRECT	**GO:0030166~proteoglycan biosynthetic process**	4.89E-02

**Database**	**Enriched pathways in COAD**	**P-Value**
GOTERM_BP_DIRECT	**GO:0016311~dephosphorylation**	1.01E-22
GOTERM_BP_DIRECT	**GO:0006508~proteolysis**	1.27E-19
KEGG_PATHWAY	**hsa04610:Complement and coagulation cascades**	1.64E-15
GOTERM_BP_DIRECT	**GO:0042730~fibrinolysis**	2.10E-08
KEGG_PATHWAY	hsa05150:Staphylococcus aureus infection	3.85E-08
GOTERM_BP_DIRECT	**GO:0006003~fructose 2,6-bisphosphate metabolic process**	6.90E-07
REACTOME_PATHWAY	**Activation of Matrix Metalloproteinases**	1.29E-06
GOTERM_BP_DIRECT	GO:0006956~complement activation	1.63E-06
KEGG_PATHWAY	hsa01100:Metabolic pathways	3.25E-06
GOTERM_BP_DIRECT	**GO:0022617~extracellular matrix disassembly**	1.60E-05
GOTERM_BP_DIRECT	**GO:0006024~glycosaminoglycan biosynthetic process**	2.75E-05
GOTERM_BP_DIRECT	**GO:0015012~heparan sulfate proteoglycan biosynthetic process**	4.52E-05
GOTERM_BP_DIRECT	**GO:0035335~peptidyl-tyrosine dephosphorylation**	5.75E-05
KEGG_PATHWAY	**hsa00534:Glycosaminoglycan biosynthesis - heparan sulfate / heparin**	7.54E-05
GOTERM_BP_DIRECT	**GO:0051919~positive regulation of fibrinolysis**	1.02E-04
GOTERM_BP_DIRECT	**GO:0015014~heparan sulfate proteoglycan biosynthetic process, polysaccharide chain biosynthetic process**	1.02E-04
GOTERM_BP_DIRECT	**GO:0048008~platelet-derived growth factor receptor signaling pathway**	2.35E-04
REACTOME_PATHWAY	Regulation of Insulin-like Growth Factor (IGF) transport and uptake by Insulin-like Growth Factor Binding Proteins (IGFBPs)	2.46E-04
REACTOME_PATHWAY	**Intrinsic Pathway of Fibrin Clot Formation**	2.83E-04
KEGG_PATHWAY	**hsa00533:Glycosaminoglycan biosynthesis - keratan sulfate**	3.70E-04
GOTERM_BP_DIRECT	**GO:0006487~protein N-linked glycosylation**	5.69E-04
GOTERM_BP_DIRECT	**GO:0006000~fructose metabolic process**	7.54E-04
GOTERM_BP_DIRECT	**GO:0006958~complement activation, classical pathway**	7.74E-04
GOTERM_BP_DIRECT	GO:0006094~gluconeogenesis	8.13E-04
GOTERM_BP_DIRECT	**GO:0043647~inositol phosphate metabolic process**	9.86E-04
REACTOME_PATHWAY	**Initial triggering of complement**	9.96E-04
GOTERM_BP_DIRECT	**GO:0007596~blood coagulation**	1.02E-03
GOTERM_BP_DIRECT	**GO:0006486~protein glycosylation**	1.27E-03
GOTERM_BP_DIRECT	GO:0006195~purine nucleotide catabolic process	1.30E-03
REACTOME_PATHWAY	R-HSA-74259:R-HSA-74259	1.85E-03

KEGG_PATHWAY	**hsa00601:Glycosphingolipid biosynthesis - lacto and neolacto series**	1.96E-03
GOTERM_BP_DIRECT	**GO:0007597~blood coagulation, intrinsic pathway**	2.51E-03
REACTOME_PATHWAY	Purine catabolism	2.60E-03
KEGG_PATHWAY	**hsa00760:Nicotinate and nicotinamide metabolism**	2.69E-03
GOTERM_BP_DIRECT	GO:0016485~protein processing	3.37E-03
KEGG_PATHWAY	**hsa00051:Fructose and mannose metabolism**	3.58E-03
GOTERM_BP_DIRECT	**GO:0046835~carbohydrate phosphorylation**	4.10E-03
GOTERM_BP_DIRECT	GO:0061621~canonical glycolysis	5.22E-03
GOTERM_BP_DIRECT	**GO:0001958~endochondral ossification**	5.22E-03
GOTERM_BP_DIRECT	**GO:0018146~keratan sulfate biosynthetic process**	6.04E-03
GOTERM_BP_DIRECT	**GO:0030449~regulation of complement activation**	6.91E-03
GOTERM_BP_DIRECT	**GO:0002542~Factor XII activation**	8.32E-03
GOTERM_BP_DIRECT	**GO:0050653~chondroitin sulfate proteoglycan biosynthetic process, polysaccharide chain biosynthetic process**	8.32E-03
GOTERM_BP_DIRECT	**GO:0033692~cellular polysaccharide biosynthetic process**	8.32E-03
GOTERM_BP_DIRECT	GO:0006096~glycolytic process	8.82E-03
REACTOME_PATHWAY	**Keratan sulfate biosynthesis**	1.19E-02
REACTOME_PATHWAY	**Regulation of Complement cascade**	1.19E-02
GOTERM_BP_DIRECT	**GO:0050651~dermatan sulfate proteoglycan biosynthetic process**	1.25E-02
GOTERM_BP_DIRECT	**GO:0019276~UDP-N-acetylgalactosamine metabolic process**	1.25E-02
GOTERM_BP_DIRECT	GO:0044267~cellular protein metabolic process	1.32E-02
GOTERM_BP_DIRECT	GO:0050900~leukocyte migration	1.44E-02
REACTOME_PATHWAY	Glycolysis	1.54E-02
GOTERM_BP_DIRECT	**GO:0006470~protein dephosphorylation**	1.57E-02
KEGG_PATHWAY	**hsa00532:Glycosaminoglycan biosynthesis - chondroitin sulfate / dermatan sulfate**	1.59E-02
GOTERM_BP_DIRECT	**GO:0006670~sphingosine metabolic process**	1.66E-02
GOTERM_BP_DIRECT	**GO:0033133~positive regulation of glucokinase activity**	2.07E-02
GOTERM_BP_DIRECT	**GO:0030311~poly-N-acetyllactosamine biosynthetic process**	2.07E-02
GOTERM_BP_DIRECT	**GO:0043456~regulation of pentose-phosphate shunt**	2.07E-02
KEGG_PATHWAY	**hsa00010:Glycolysis / Gluconeogenesis**	2.72E-02
GOTERM_BP_DIRECT	GO:0007586~digestion	2.84E-02
GOTERM_BP_DIRECT	**GO:0007411~axon guidance**	2.87E-02
GOTERM_BP_DIRECT	**GO:0001867~complement activation, lectin pathway**	2.88E-02
REACTOME_PATHWAY	**Alternative complement activation**	2.94E-02
REACTOME_PATHWAY	Ficolins bind to repetitive carbohydrate structures on the target cell surface	2.94E-02
KEGG_PATHWAY	**hsa04152:AMPK signaling pathway**	3.14E-02
KEGG_PATHWAY	**hsa00562:Inositol phosphate metabolism**	3.15E-02
GOTERM_BP_DIRECT	GO:0014909~smooth muscle cell migration	3.29E-02
GOTERM_BP_DIRECT	**GO:0050650~chondroitin sulfate proteoglycan biosynthetic process**	3.29E-02
KEGG_PATHWAY	**hsa00052:Galactose metabolism**	3.41E-02
REACTOME_PATHWAY	**Lectin pathway of complement activation**	3.52E-02
GOTERM_BP_DIRECT	**GO:0005975~carbohydrate metabolic process**	3.61E-02
GOTERM_BP_DIRECT	**GO:0031638~zymogen activation**	3.69E-02

GOTERM_BP_DIRECT	**GO:0031639~plasminogen activation**	3.69E-02
GOTERM_BP_DIRECT	GO:0050778~positive regulation of immune response	3.69E-02
KEGG_PATHWAY	**hsa00500:Starch and sucrose metabolism**	4.06E-02
GOTERM_BP_DIRECT	**GO:0051918~negative regulation of fibrinolysis**	4.09E-02
GOTERM_BP_DIRECT	**GO:0042355~L-fucose catabolic process**	4.09E-02
GOTERM_BP_DIRECT	**GO:0051156~glucose 6-phosphate metabolic process**	4.09E-02
KEGG_PATHWAY	hsa05322:Systemic lupus erythematosus	4.11E-02
GOTERM_BP_DIRECT	GO:0050728~negative regulation of inflammatory response	4.30E-02
GOTERM_BP_DIRECT	**GO:0001503~ossification**	4.39E-02
GOTERM_BP_DIRECT	GO:0046135~pyrimidine nucleoside catabolic process	4.49E-02
GOTERM_BP_DIRECT	**GO:0046855~inositol phosphate dephosphorylation**	4.49E-02
GOTERM_BP_DIRECT	**GO:0033628~regulation of cell adhesion mediated by integrin**	4.49E-02
GOTERM_BP_DIRECT	**GO:0017187~peptidyl-glutamic acid carboxylation**	4.49E-02
GOTERM_BP_DIRECT	GO:0002003~angiotensin maturation	4.49E-02
GOTERM_BP_DIRECT	**GO:0009312~oligosaccharide biosynthetic process**	4.49E-02
REACTOME_PATHWAY	**Activation of C3 and C5**	4.66E-02
GOTERM_BP_DIRECT	**GO:0030166~proteoglycan biosynthetic process**	4.89E-02

Database	Enriched pathways in ESCA	P-Value
KEGG_PATHWAY	hsa01100:Metabolic pathways	2.47E-12
KEGG_PATHWAY	**hsa00601:Glycosphingolipid biosynthesis - lacto and neolacto series**	8.77E-10
GOTERM_BP_DIRECT	**GO:0018146~keratan sulfate biosynthetic process**	1.22E-09
KEGG_PATHWAY	**hsa00533:Glycosaminoglycan biosynthesis - keratan sulfate**	3.24E-09
REACTOME_PATHWAY	**Keratan sulfate biosynthesis**	6.21E-09
GOTERM_BP_DIRECT	**GO:0006486~protein glycosylation**	4.23E-08
GOTERM_BP_DIRECT	**GO:0009312~oligosaccharide biosynthetic process**	8.41E-07
KEGG_PATHWAY	**hsa00510:N-Glycan biosynthesis**	1.85E-06
REACTOME_PATHWAY	**N-Glycan antennae elongation**	6.34E-06
GOTERM_BP_DIRECT	**GO:0032259~methylation**	8.27E-06
GOTERM_BP_DIRECT	**GO:0046500~S-adenosylmethionine metabolic process**	6.44E-05
GOTERM_BP_DIRECT	**GO:0015012~heparan sulfate proteoglycan biosynthetic process**	4.13E-04
KEGG_PATHWAY	**hsa01212:Fatty acid metabolism**	1.13E-03
GOTERM_BP_DIRECT	**GO:0006487~protein N-linked glycosylation**	2.19E-03
GOTERM_BP_DIRECT	**GO:0006024~glycosaminoglycan biosynthetic process**	2.54E-03
GOTERM_BP_DIRECT	**GO:0005975~carbohydrate metabolic process**	3.61E-03
KEGG_PATHWAY	**hsa00514:Other types of O-glycan biosynthesis**	4.02E-03
KEGG_PATHWAY	**hsa00534:Glycosaminoglycan biosynthesis - heparan sulfate / heparin**	4.78E-03
GOTERM_BP_DIRECT	**GO:0016266~O-glycan processing**	5.12E-03
GOTERM_BP_DIRECT	**GO:0046498~S-adenosylhomocysteine metabolic process**	7.13E-03
REACTOME_PATHWAY	**O-linked glycosylation of mucins**	9.63E-03
GOTERM_BP_DIRECT	**GO:0006853~carnitine shuttle**	1.60E-02
GOTERM_BP_DIRECT	**GO:0036109~alpha-linolenic acid metabolic process**	2.30E-02
REACTOME_PATHWAY	Import of palmitoyl-CoA into the mitochondrial matrix	2.75E-02
REACTOME_PATHWAY	**alpha-linolenic acid (ALA) metabolism**	3.25E-02
KEGG_PATHWAY	**hsa03320:PPAR signaling pathway**	3.41E-02
GOTERM_BP_DIRECT	**GO:0030206~chondroitin sulfate biosynthetic process**	4.38E-02
GOTERM_BP_DIRECT	**GO:0046487~glyoxylate metabolic process**	4.55E-02
KEGG_PATHWAY	**hsa00130:Ubiquinone and other terpenoid-quinone biosynthesis**	4.70E-02
REACTOME_PATHWAY	Signaling by Retinoic Acid	4.95E-02
REACTOME_PATHWAY	**Chondroitin sulfate biosynthesis**	4.95E-02

Database	Enriched pathways in HNSC	P-Value
GOTERM_BP_DIRECT	**GO:0016310~phosphorylation**	1.21E-17
GOTERM_BP_DIRECT	**GO:0046835~carbohydrate phosphorylation**	2.46E-17
KEGG_PATHWAY	hsa01100:Metabolic pathways	2.09E-16
GOTERM_BP_DIRECT	**GO:0006661~phosphatidylinositol biosynthetic process**	2.16E-14
KEGG_PATHWAY	**hsa00562:Inositol phosphate metabolism**	2.74E-13
KEGG_PATHWAY	**hsa04070:Phosphatidylinositol signaling system**	1.80E-12
REACTOME_PATHWAY	Synthesis of PIPs at the plasma membrane	3.26E-10
GOTERM_BP_DIRECT	**GO:0006508~proteolysis**	3.99E-10
GOTERM_BP_DIRECT	**GO:0046854~phosphatidylinositol phosphorylation**	3.17E-09

KEGG_PATHWAY	**hsa00051:Fructose and mannose metabolism**	6.08E-09
GOTERM_BP_DIRECT	**GO:0036092~phosphatidylinositol-3-phosphate biosynthetic process**	1.13E-08
GOTERM_BP_DIRECT	**GO:0016311~dephosphorylation**	3.12E-08
GOTERM_BP_DIRECT	**GO:0006002~fructose 6-phosphate metabolic process**	6.90E-08
KEGG_PATHWAY	**hsa00010:Glycolysis / Gluconeogenesis**	2.08E-07
KEGG_PATHWAY	hsa01200:Carbon metabolism	2.53E-07
KEGG_PATHWAY	**hsa04610:Complement and coagulation cascades**	2.70E-07
GOTERM_BP_DIRECT	GO:0006096~glycolytic process	1.34E-06
KEGG_PATHWAY	hsa05230:Central carbon metabolism in cancer	1.80E-06
GOTERM_BP_DIRECT	**GO:0030388~fructose 1,6-bisphosphate metabolic process**	6.23E-06
GOTERM_BP_DIRECT	**GO:0005975~carbohydrate metabolic process**	6.91E-06
GOTERM_BP_DIRECT	**GO:0043647~inositol phosphate metabolic process**	6.97E-06
GOTERM_BP_DIRECT	**GO:0006470~protein dephosphorylation**	7.76E-06
GOTERM_BP_DIRECT	GO:0061621~canonical glycolysis	1.36E-05
KEGG_PATHWAY	hsa01130:Biosynthesis of antibiotics	2.22E-05
KEGG_PATHWAY	**hsa00030:Pentose phosphate pathway**	3.53E-05
KEGG_PATHWAY	**hsa00052:Galactose metabolism**	4.20E-05
REACTOME_PATHWAY	Synthesis of IP2, IP, and Ins in the cytosol	8.88E-05
REACTOME_PATHWAY	Glycolysis	1.37E-04
REACTOME_PATHWAY	**Activation of Matrix Metalloproteinases**	1.55E-04
GOTERM_BP_DIRECT	**GO:0042730~fibrinolysis**	2.23E-04
GOTERM_BP_DIRECT	**GO:0061624~fructose catabolic process to hydroxyacetone phosphate and glyceraldehyde-3-phosphate**	3.20E-04
GOTERM_BP_DIRECT	GO:0009157~deoxyribonucleoside monophosphate biosynthetic process	3.20E-04
KEGG_PATHWAY	**hsa00601:Glycosphingolipid biosynthesis - lacto and neolacto series**	3.58E-04
REACTOME_PATHWAY	Synthesis of PIPs at the Golgi membrane	4.21E-04
GOTERM_BP_DIRECT	**GO:0006486~protein glycosylation**	4.74E-04
REACTOME_PATHWAY	**Fructose catabolism**	6.81E-04
REACTOME_PATHWAY	**Intrinsic Pathway of Fibrin Clot Formation**	7.75E-04
GOTERM_BP_DIRECT	**GO:0022617~extracellular matrix disassembly**	9.39E-04
GOTERM_BP_DIRECT	**GO:0006000~fructose metabolic process**	1.41E-03
GOTERM_BP_DIRECT	**GO:0046855~inositol phosphate dephosphorylation**	1.72E-03
GOTERM_BP_DIRECT	**GO:0017187~peptidyl-glutamic acid carboxylation**	1.72E-03
GOTERM_BP_DIRECT	**GO:0006071~glycerol metabolic process**	1.72E-03
REACTOME_PATHWAY	Transport of gamma-carboxylated protein precursors from the endoplasmic reticulum to the Golgi apparatus	2.40E-03
GOTERM_BP_DIRECT	**GO:0035335~peptidyl-tyrosine dephosphorylation**	2.49E-03
REACTOME_PATHWAY	Gamma-carboxylation of protein precursors	2.98E-03
REACTOME_PATHWAY	Removal of aminoterminal propeptides from gamma-carboxylated proteins	2.98E-03
REACTOME_PATHWAY	Synthesis of PIPs at the late endosome membrane	2.98E-03
GOTERM_BP_DIRECT	**GO:0048015~phosphatidylinositol-mediated signaling**	3.19E-03
GOTERM_BP_DIRECT	**GO:0009165~nucleotide biosynthetic process**	3.24E-03
KEGG_PATHWAY	**hsa00520:Amino sugar and nucleotide sugar metabolism**	3.72E-03
KEGG_PATHWAY	hsa04930:Type II diabetes mellitus	3.72E-03

GOTERM_BP_DIRECT	GO:0006796~phosphate-containing compound metabolic process	4.66E-03
GOTERM_BP_DIRECT	**GO:0007597~blood coagulation, intrinsic pathway**	4.66E-03
KEGG_PATHWAY	**hsa04152:AMPK signaling pathway**	5.90E-03
REACTOME_PATHWAY	Synthesis of PIPs at the early endosome membrane	5.90E-03
KEGG_PATHWAY	**hsa00760:Nicotinate and nicotinamide metabolism**	6.58E-03
GOTERM_BP_DIRECT	GO:0006006~glucose metabolic process	6.66E-03
GOTERM_BP_DIRECT	GO:0016485~protein processing	8.12E-03
GOTERM_BP_DIRECT	**GO:0046856~phosphatidylinositol dephosphorylation**	8.23E-03
GOTERM_BP_DIRECT	GO:0006465~signal peptide processing	8.91E-03
GOTERM_BP_DIRECT	**GO:0014066~regulation of phosphatidylinositol 3-kinase signaling**	1.01E-02
KEGG_PATHWAY	hsa04910:Insulin signaling pathway	1.02E-02
GOTERM_BP_DIRECT	GO:0050728~negative regulation of inflammatory response	1.05E-02
GOTERM_BP_DIRECT	**GO:0018146~keratan sulfate biosynthetic process**	1.11E-02
GOTERM_BP_DIRECT	**GO:0050653~chondroitin sulfate proteoglycan biosynthetic process, polysaccharide chain biosynthetic process**	1.14E-02
GOTERM_BP_DIRECT	GO:0046104~thymidine metabolic process	1.14E-02
GOTERM_BP_DIRECT	**GO:0006001~fructose catabolic process**	1.14E-02
GOTERM_BP_DIRECT	**GO:0005986~sucrose biosynthetic process**	1.14E-02
REACTOME_PATHWAY	Regulation of Insulin-like Growth Factor (IGF) transport and uptake by Insulin-like Growth Factor Binding Proteins (IGFBPs)	1.31E-02
GOTERM_BP_DIRECT	**GO:0006956~complement activation**	1.36E-02
REACTOME_PATHWAY	**Common Pathway of Fibrin Clot Formation**	1.43E-02
KEGG_PATHWAY	**hsa01230:Biosynthesis of amino acids**	1.54E-02
KEGG_PATHWAY	**hsa00533:Glycosaminoglycan biosynthesis - keratan sulfate**	1.66E-02
GOTERM_BP_DIRECT	**GO:0050651~dermatan sulfate proteoglycan biosynthetic process**	1.71E-02
GOTERM_BP_DIRECT	**GO:0046167~glycerol-3-phosphate biosynthetic process**	1.71E-02
GOTERM_BP_DIRECT	**GO:0007596~blood coagulation**	2.12E-02
GOTERM_BP_DIRECT	GO:1901215~negative regulation of neuron death	2.19E-02
GOTERM_BP_DIRECT	**GO:0006670~sphingosine metabolic process**	2.27E-02
GOTERM_BP_DIRECT	**GO:0006021~inositol biosynthetic process**	2.27E-02
REACTOME_PATHWAY	**Keratan sulfate biosynthesis**	2.27E-02
KEGG_PATHWAY	**hsa00600:Sphingolipid metabolism**	2.46E-02
GOTERM_BP_DIRECT	**GO:0006094~gluconeogenesis**	2.62E-02
GOTERM_BP_DIRECT	**GO:0043456~regulation of pentose-phosphate shunt**	2.83E-02
REACTOME_PATHWAY	Synthesis of IPs in the nucleus	3.31E-02
GOTERM_BP_DIRECT	**GO:0032958~inositol phosphate biosynthetic process**	3.38E-02
GOTERM_BP_DIRECT	**GO:0019563~glycerol catabolic process**	3.38E-02
KEGG_PATHWAY	hsa05150:Staphylococcus aureus infection	3.52E-02
GOTERM_BP_DIRECT	**GO:0001867~complement activation, lectin pathway**	3.93E-02
GOTERM_BP_DIRECT	**GO:0006020~inositol metabolic process**	3.93E-02
REACTOME_PATHWAY	Ficolins bind to repetitive carbohydrate structures on the target cell surface	4.12E-02
REACTOME_PATHWAY	Glucose metabolism	4.12E-02
KEGG_PATHWAY	**hsa00561:Glycerolipid metabolism**	4.21E-02
GOTERM_BP_DIRECT	GO:0014909~smooth muscle cell migration	4.48E-02

GOTERM_BP_DIRECT	**GO:0050650~chondroitin sulfate proteoglycan biosynthetic process**	4.48E-02
GOTERM_BP_DIRECT	GO:0051289~protein homotetramerization	4.62E-02
REACTOME_PATHWAY	**Lectin pathway of complement activation**	4.92E-02

**Database**	**Enriched pathways in KICH**	**P-Value**
KEGG_PATHWAY	hsa01100:Metabolic pathways	5.25E-08
KEGG_PATHWAY	**hsa00270:Cysteine and methionine metabolism**	3.80E-07
GOTERM_BP_DIRECT	**GO:0090116~C-5 methylation of cytosine**	1.40E-06
KEGG_PATHWAY	**hsa00520:Amino sugar and nucleotide sugar metabolism**	6.71E-05
GOTERM_BP_DIRECT	**GO:0006306~DNA methylation**	1.39E-04
GOTERM_BP_DIRECT	GO:0045814~negative regulation of gene expression, epigenetic	5.63E-04
GOTERM_BP_DIRECT	**GO:0046499~S-adenosylmethioninamine metabolic process**	1.43E-03
GOTERM_BP_DIRECT	**GO:0010424~DNA methylation on cytosine within a CG sequence**	2.14E-03
REACTOME_PATHWAY	**DNA methylation**	2.67E-03
GOTERM_BP_DIRECT	**GO:0046498~S-adenosylhomocysteine metabolic process**	2.86E-03
REACTOME_PATHWAY	PRC2 methylates histones and DNA	3.35E-03
GOTERM_BP_DIRECT	**GO:0043045~DNA methylation involved in embryo development**	3.57E-03
GOTERM_BP_DIRECT	GO:0051573~negative regulation of histone H3-K9 methylation	4.28E-03
REACTOME_PATHWAY	**Metabolism of polyamines**	4.84E-03
GOTERM_BP_DIRECT	**GO:0006595~polyamine metabolic process**	4.99E-03
GOTERM_BP_DIRECT	**GO:0006048~UDP-N-acetylglucosamine biosynthetic process**	7.84E-03
GOTERM_BP_DIRECT	**GO:0051571~positive regulation of histone H3-K4 methylation**	1.21E-02
GOTERM_BP_DIRECT	**GO:0033189~response to vitamin A**	1.28E-02
GOTERM_BP_DIRECT	**GO:0042220~response to cocaine**	2.26E-02
GOTERM_BP_DIRECT	**GO:0071230~cellular response to amino acid stimulus**	3.31E-02
GOTERM_BP_DIRECT	**GO:0010212~response to ionizing radiation**	3.45E-02

**database**	**Enriched pathways in KIRC**	**P-Value**
GOTERM_BP_DIRECT	**GO:0005975~carbohydrate metabolic process**	8.13E-28
GOTERM_BP_DIRECT	**GO:0016311~dephosphorylation**	1.55E-17
KEGG_PATHWAY	hsa01100:Metabolic pathways	7.47E-13
GOTERM_BP_DIRECT	**GO:0006470~protein dephosphorylation**	6.07E-11
REACTOME_PATHWAY	Synthesis of IP2, IP, and Ins in the cytosol	8.28E-11
KEGG_PATHWAY	**hsa00511:Other glycan degradation**	9.76E-10
GOTERM_BP_DIRECT	**GO:0043647~inositol phosphate metabolic process**	4.89E-09
KEGG_PATHWAY	**hsa04142:Lysosome**	2.25E-07
GOTERM_BP_DIRECT	**GO:0046855~inositol phosphate dephosphorylation**	2.36E-07
GOTERM_BP_DIRECT	**GO:0035335~peptidyl-tyrosine dephosphorylation**	9.27E-07
GOTERM_BP_DIRECT	**GO:0016139~glycoside catabolic process**	1.43E-06
GOTERM_BP_DIRECT	GO:0006796~phosphate-containing compound metabolic process	2.13E-06
KEGG_PATHWAY	**hsa00562:Inositol phosphate metabolism**	2.20E-06
KEGG_PATHWAY	**hsa00531:Glycosaminoglycan degradation**	2.60E-06

GOTERM_BP_DIRECT	**GO:0006687~glycosphingolipid metabolic process**	3.86E-06
GOTERM_BP_DIRECT	**GO:0006027~glycosaminoglycan catabolic process**	1.01E-05
GOTERM_BP_DIRECT	**GO:0006661~phosphatidylinositol biosynthetic process**	1.37E-05
KEGG_PATHWAY	**hsa00600:Sphingolipid metabolism**	2.13E-05
GOTERM_BP_DIRECT	**GO:0009313~oligosaccharide catabolic process**	2.30E-05
REACTOME_PATHWAY	**Glycosphingolipid metabolism**	2.44E-05
KEGG_PATHWAY	**hsa04070:Phosphatidylinositol signaling system**	2.48E-05
REACTOME_PATHWAY	Digestion of dietary carbohydrate	3.73E-05
KEGG_PATHWAY	hsa00500:Starch and sucrose metabolism	4.63E-05
GOTERM_BP_DIRECT	**GO:0016042~lipid catabolic process**	8.77E-05
REACTOME_PATHWAY	Synthesis of PIPs at the plasma membrane	1.50E-04
GOTERM_BP_DIRECT	**GO:0044245~polysaccharide digestion**	1.66E-04
KEGG_PATHWAY	**hsa04610:Complement and coagulation cascades**	1.92E-04
GOTERM_BP_DIRECT	**GO:0046856~phosphatidylinositol dephosphorylation**	2.69E-04
GOTERM_BP_DIRECT	**GO:0006508~proteolysis**	3.10E-04
KEGG_PATHWAY	**hsa00052:Galactose metabolism**	4.66E-04
GOTERM_BP_DIRECT	**GO:0030388~fructose 1,6-bisphosphate metabolic process**	5.73E-04
REACTOME_PATHWAY	**HS-GAG degradation**	6.35E-04
GOTERM_BP_DIRECT	**GO:0006517~protein deglycosylation**	1.22E-03
GOTERM_BP_DIRECT	**GO:0036092~phosphatidylinositol-3-phosphate biosynthetic process**	2.22E-03
REACTOME_PATHWAY	**Dissolution of Fibrin Clot**	4.45E-03
KEGG_PATHWAY	hsa01130:Biosynthesis of antibiotics	4.48E-03
KEGG_PATHWAY	**hsa00030:Pentose phosphate pathway**	5.28E-03
GOTERM_BP_DIRECT	**GO:0042730~fibrinolysis**	5.46E-03
REACTOME_PATHWAY	**N-glycan trimming in the ER and Calnexin/Calreticulin cycle**	6.75E-03
GOTERM_BP_DIRECT	**GO:0036151~phosphatidylcholine acyl-chain remodeling**	8.95E-03
GOTERM_BP_DIRECT	**GO:0006629~lipid metabolic process**	9.71E-03
GOTERM_BP_DIRECT	**GO:0046477~glycosylceramide catabolic process**	1.06E-02
GOTERM_BP_DIRECT	**GO:0005986~sucrose biosynthetic process**	1.06E-02
GOTERM_BP_DIRECT	**GO:0006001~fructose catabolic process**	1.06E-02
REACTOME_PATHWAY	Regulation of Insulin-like Growth Factor (IGF) transport and uptake by Insulin-like Growth Factor Binding Proteins (IGFBPs)	1.15E-02
KEGG_PATHWAY	hsa01200:Carbon metabolism	1.27E-02
GOTERM_BP_DIRECT	**GO:0046854~phosphatidylinositol phosphorylation**	1.36E-02
GOTERM_BP_DIRECT	**GO:0043407~negative regulation of MAP kinase activity**	1.56E-02
GOTERM_BP_DIRECT	**GO:0007040~lysosome organization**	1.56E-02
REACTOME_PATHWAY	Intestinal saccharidase deficiencies	1.56E-02
GOTERM_BP_DIRECT	**GO:0000023~maltose metabolic process**	1.58E-02
KEGG_PATHWAY	**hsa00565:Ether lipid metabolism**	1.78E-02
REACTOME_PATHWAY	Acyl chain remodelling of PC	1.87E-02
GOTERM_BP_DIRECT	GO:1901215~negative regulation of neuron death	1.90E-02
REACTOME_PATHWAY	Synthesis of IP3 and IP4 in the cytosol	2.00E-02
GOTERM_BP_DIRECT	**GO:0030200~heparan sulfate proteoglycan catabolic process**	2.10E-02

GOTERM_BP_DIRECT	**GO:0006021~inositol biosynthetic process**	2.10E-02
GOTERM_BP_DIRECT	**GO:0006670~sphingosine metabolic process**	2.10E-02
GOTERM_BP_DIRECT	**GO:0006094~gluconeogenesis**	2.27E-02
GOTERM_BP_DIRECT	**GO:0043456~regulation of pentose-phosphate shunt**	2.62E-02
REACTOME_PATHWAY	**Sialic acid metabolism**	2.73E-02
REACTOME_PATHWAY	**Activation of Matrix Metalloproteinases**	2.73E-02
KEGG_PATHWAY	hsa05150:Staphylococcus aureus infection	2.87E-02
GOTERM_BP_DIRECT	**GO:0006689~ganglioside catabolic process**	3.14E-02
KEGG_PATHWAY	**hsa00561:Glycerolipid metabolism**	3.45E-02
GOTERM_BP_DIRECT	GO:0009056~catabolic process	3.65E-02
GOTERM_BP_DIRECT	**GO:0006013~mannose metabolic process**	3.65E-02
GOTERM_BP_DIRECT	**GO:0006020~inositol metabolic process**	3.65E-02
GOTERM_BP_DIRECT	**GO:0006004~fucose metabolic process**	3.65E-02
GOTERM_BP_DIRECT	**GO:0006032~chitin catabolic process**	3.65E-02
GOTERM_BP_DIRECT	GO:0048771~tissue remodeling	3.65E-02
KEGG_PATHWAY	**hsa00592:alpha-Linolenic acid metabolis**m	3.77E-02
GOTERM_BP_DIRECT	GO:0007416~synapse assembly	4.15E-02
GOTERM_BP_DIRECT	GO:0014909~smooth muscle cell migration	4.16E-02
GOTERM_BP_DIRECT	**GO:0006002~fructose 6-phosphate metabolic process**	4.16E-02
GOTERM_BP_DIRECT	**GO:0030299~intestinal cholesterol absorption**	4.67E-02
GOTERM_BP_DIRECT	**GO:0006006~glucose metabolic process**	4.92E-02
KEGG_PATHWAY	**hsa00591:Linoleic acid metabolism**	4.94E-02

**Database**	**Enriched pathways in KIRP**	**P-Value**
GOTERM_BP_DIRECT	**GO:0016311~dephosphorylation**	7.70E-14
KEGG_PATHWAY	hsa04972:Pancreatic secretion	2.10E-13
GOTERM_BP_DIRECT	**GO:0006508~proteolysis**	2.34E-12
GOTERM_BP_DIRECT	**GO:0016042~lipid catabolic process**	2.66E-12
KEGG_PATHWAY	**hsa04610:Complement and coagulation cascades**	5.22E-11
GOTERM_BP_DIRECT	**GO:0006629~lipid metabolic process**	1.25E-09
GOTERM_BP_DIRECT	**GO:0042730~fibrinolysis**	2.43E-08
REACTOME_PATHWAY	Digestion of dietary lipid	5.22E-08
GOTERM_BP_DIRECT	GO:0015991~ATP hydrolysis coupled proton transport	2.32E-07
KEGG_PATHWAY	**hsa00561:Glycerolipid metabolism**	1.50E-06
REACTOME_PATHWAY	**Activation of Matrix Metalloproteinases**	1.85E-06
KEGG_PATHWAY	hsa01100:Metabolic pathways	1.89E-06
KEGG_PATHWAY	**hsa04975:Fat digestion and absorption**	2.05E-06
GOTERM_BP_DIRECT	**GO:0022617~extracellular matrix disassembly**	1.84E-05
GOTERM_BP_DIRECT	**GO:0044241~lipid digestion**	2.10E-05
GOTERM_BP_DIRECT	GO:0034220~ion transmembrane transport	3.20E-05
GOTERM_BP_DIRECT	**GO:0007597~blood coagulation, intrinsic pathway**	5.89E-05
KEGG_PATHWAY	**hsa00565:Ether lipid metabolism**	7.85E-05
GOTERM_BP_DIRECT	**GO:0051919~positive regulation of fibrinolysis**	1.08E-04

GOTERM_BP_DIRECT	GO:0015986~ATP synthesis coupled proton transport	1.10E-04
GOTERM_BP_DIRECT	GO:0007586~digestion	1.53E-04
KEGG_PATHWAY	hsa05150:Staphylococcus aureus infection	1.89E-04
GOTERM_BP_DIRECT	**GO:0036151~phosphatidylcholine acyl-chain remodeling**	2.05E-04
REACTOME_PATHWAY	**Intrinsic Pathway of Fibrin Clot Formation**	3.50E-04
KEGG_PATHWAY	**hsa00564:Glycerophospholipid metabolism**	3.52E-04
GOTERM_BP_DIRECT	**GO:0030388~fructose 1,6-bisphosphate metabolic process**	3.75E-04
GOTERM_BP_DIRECT	**GO:0006641~triglyceride metabolic process**	4.48E-04
REACTOME_PATHWAY	Ion transport by P-type ATPases	4.61E-04
REACTOME_PATHWAY	Acyl chain remodelling of PC	6.50E-04
GOTERM_BP_DIRECT	GO:0007035~vacuolar acidification	1.60E-03
GOTERM_BP_DIRECT	**GO:0006644~phospholipid metabolic process**	1.60E-03
GOTERM_BP_DIRECT	GO:1903779~regulation of cardiac conduction	1.78E-03
KEGG_PATHWAY	hsa04974:Protein digestion and absorption	1.80E-03
KEGG_PATHWAY	**hsa00592:alpha-Linolenic acid metabolism**	1.90E-03
KEGG_PATHWAY	hsa00190:Oxidative phosphorylation	2.06E-03
KEGG_PATHWAY	hsa04966:Collecting duct acid secretion	2.38E-03
GOTERM_BP_DIRECT	**GO:0036148~phosphatidylglycerol acyl-chain remodeling**	2.65E-03
KEGG_PATHWAY	**hsa00030:Pentose phosphate pathway**	2.93E-03
KEGG_PATHWAY	**hsa00591:Linoleic acid metabolism**	2.93E-03
REACTOME_PATHWAY	**Dissolution of Fibrin Clot**	2.99E-03
GOTERM_BP_DIRECT	GO**:0036152~phosphatidylethanolamine acyl-chain remodeling**	4.71E-03
REACTOME_PATHWAY	Ion homeostasis	5.40E-03
GOTERM_BP_DIRECT	GO:0090383~phagosome acidification	5.94E-03
GOTERM_BP_DIRECT	**GO:0006956~complement activation**	6.19E-03
REACTOME_PATHWAY	Acyl chain remodelling of PG	6.40E-03
KEGG_PATHWAY	**hsa04142:Lysosome**	7.11E-03
REACTOME_PATHWAY	Acyl chain remodelling of PG	7.80E-03
GOTERM_BP_DIRECT	**GO:0007596~blood coagulation**	8.04E-03
GOTERM_BP_DIRECT	**GO:0005986~sucrose biosynthetic process**	8.56E-03
GOTERM_BP_DIRECT	**GO:0002542~Factor XII activation**	8.56E-03
GOTERM_BP_DIRECT	**GO:0006001~fructose catabolic process**	8.56E-03
GOTERM_BP_DIRECT	GO:0033572~transferrin transport	9.85E-03
REACTOME_PATHWAY	Ion channel transport	1.01E-02
REACTOME_PATHWAY	Insulin receptor recycling	1.18E-02
GOTERM_BP_DIRECT	**GO:1903416~response to glycoside**	1.28E-02
REACTOME_PATHWAY	Transferrin endocytosis and recycling	1.46E-02
KEGG_PATHWAY	hsa05110:Vibrio cholerae infection	1.50E-02
GOTERM_BP_DIRECT	**GO:0006094~gluconeogenesis**	1.53E-02
REACTOME_PATHWAY	Acyl chain remodelling of PE	1.56E-02
GOTERM_BP_DIRECT	**GO:0002084~protein depalmitoylation**	1.70E-02
GOTERM_BP_DIRECT	**GO:0006670~sphingosine metabolic process**	1.70E-02
REACTOME_PATHWAY	**ROS, RNS production in phagocytes**	1.98E-02

GOTERM_BP_DIRECT	**GO:0006633~fatty acid biosynthetic process**	2.09E-02
GOTERM_BP_DIRECT	**GO:0043456~regulation of pentose-phosphate shunt**	2.13E-02
KEGG_PATHWAY	**hsa00590:Arachidonic acid metabolism**	2.30E-02
KEGG_PATHWAY	hsa01200:Carbon metabolism	2.64E-02
GOTERM_BP_DIRECT	**GO:0001523~retinoid metabolic process**	2.82E-02
GOTERM_BP_DIRECT	GO:0045822~negative regulation of heart contraction	2.96E-02
GOTERM_BP_DIRECT	GO:0048771~tissue remodeling	2.96E-02
REACTOME_PATHWAY	**Retinoid metabolism and transport**	3.07E-02
REACTOME_PATHWAY	**Alternative complement activation**	3.16E-02
GOTERM_BP_DIRECT	**GO:0006002~fructose 6-phosphate metabolic process**	3.38E-02
GOTERM_BP_DIRECT	GO:0014909~smooth muscle cell migration	3.38E-02
KEGG_PATHWAY	hsa04971:Gastric acid secretion	3.65E-02
GOTERM_BP_DIRECT	**GO:0031638~zymogen activation**	3.79E-02
GOTERM_BP_DIRECT	GO:1990573~potassium ion import across plasma membrane	3.79E-02
GOTERM_BP_DIRECT	**GO:0031639~plasminogen activation**	3.79E-02
GOTERM_BP_DIRECT	GO:0086009~membrane repolarization	3.79E-02
GOTERM_BP_DIRECT	GO:0050778~positive regulation of immune response	3.79E-02
GOTERM_BP_DIRECT	**GO:0030299~intestinal cholesterol absorption**	3.79E-02
KEGG_PATHWAY	**hsa00051:Fructose and mannose metabolism**	4.05E-02
GOTERM_BP_DIRECT	GO:0070072~vacuolar proton-transporting V-type ATPase complex assembly	4.21E-02
GOTERM_BP_DIRECT	**GO:0051918~negative regulation of fibrinolysis**	4.21E-02
GOTERM_BP_DIRECT	**GO:0006000~fructose metabolic process**	4.21E-02
GOTERM_BP_DIRECT	GO:0036376~sodium ion export from cell	4.21E-02
GOTERM_BP_DIRECT	**GO:0071383~cellular response to steroid hormone stimulus**	4.21E-02
GOTERM_BP_DIRECT	GO:0040011~locomotion	4.21E-02
GOTERM_BP_DIRECT	GO:0008286~insulin receptor signaling pathway	4.42E-02
GOTERM_BP_DIRECT	**GO:0045820~negative regulation of glycolytic process**	4.62E-02
GOTERM_BP_DIRECT	**GO:0006098~pentose-phosphate shunt**	4.62E-02
GOTERM_BP_DIRECT	GO:0060081~membrane hyperpolarization	4.62E-02
GOTERM_BP_DIRECT	**GO:0017187~peptidyl-glutamic acid carboxylation**	4.62E-02

**Database**	**Enriched pathways in LIHC**	**P-Value**
GOTERM_BP_DIRECT	**GO:0016311~dephosphorylation**	2.53E-16
KEGG_PATHWAY	hsa01100:Metabolic pathways	9.27E-15
KEGG_PATHWAY	**hsa00601:Glycosphingolipid biosynthesis - lacto and neolacto series**	3.28E-11
GOTERM_BP_DIRECT	**GO:0006486~protein glycosylation**	8.18E-09
GOTERM_BP_DIRECT	**GO:0030148~sphingolipid biosynthetic process**	8.82E-07
GOTERM_BP_DIRECT	**GO:0035335~peptidyl-tyrosine dephosphorylation**	1.70E-06
GOTERM_BP_DIRECT	**GO:0006470~protein dephosphorylation**	6.90E-06
KEGG_PATHWAY	**hsa00533:Glycosaminoglycan biosynthesis - keratan sulfate**	7.10E-06
GOTERM_BP_DIRECT	**GO:0009312~oligosaccharide biosynthetic process**	7.74E-06
KEGG_PATHWAY	**hsa00514:Other types of O-glycan biosynthesis**	3.63E-05

GOTERM_BP_DIRECT	**GO:0005975~carbohydrate metabolic process**	4.32E-05
REACTOME_PATHWAY	**Sphingolipid de novo biosynthesis**	5.10E-05
REACTOME_PATHWAY	**N-Glycan antennae elongation**	5.31E-05
GOTERM_BP_DIRECT	**GO:0030206~chondroitin sulfate biosynthetic process**	1.04E-04
REACTOME_PATHWAY	**Chondroitin sulfate biosynthesis**	1.31E-04
GOTERM_BP_DIRECT	**GO:0018146~keratan sulfate biosynthetic process**	1.47E-04
GOTERM_BP_DIRECT	**GO:0030388~fructose 1,6-bisphosphate metabolic process**	2.78E-04
REACTOME_PATHWAY	**Keratan sulfate biosynthesis**	3.65E-04
GOTERM_BP_DIRECT	**GO:0006487~protein N-linked glycosylation**	3.98E-04
GOTERM_BP_DIRECT	**GO:0042355~L-fucose catabolic process**	5.92E-04
KEGG_PATHWAY	**hsa00532:Glycosaminoglycan biosynthesis - chondroitin sulfate / dermatan sulfate**	6.79E-04
GOTERM_BP_DIRECT	**GO:0046839~phospholipid dephosphorylation**	7.22E-04
KEGG_PATHWAY	**hsa00600:Sphingolipid metabolism**	7.51E-04
GOTERM_BP_DIRECT	**GO:0006644~phospholipid metabolic process**	1.04E-03
GOTERM_BP_DIRECT	**GO:0015012~heparan sulfate proteoglycan biosynthetic process**	1.76E-03
GOTERM_BP_DIRECT	**GO:0006508~proteolysis**	2.38E-03
KEGG_PATHWAY	hsa01130:Biosynthesis of antibiotics	2.54E-03
KEGG_PATHWAY	**hsa04610:Complement and coagulation cascades**	3.15E-03
KEGG_PATHWAY	**hsa00565:Ether lipid metabolism**	7.23E-03
GOTERM_BP_DIRECT	**GO:0050653~chondroitin sulfate proteoglycan biosynthetic process, polysaccharide chain biosynthetic process**	7.37E-03
GOTERM_BP_DIRECT	**GO:0006001~fructose catabolic process**	7.37E-03
GOTERM_BP_DIRECT	**GO:0005986~sucrose biosynthetic process**	7.37E-03
KEGG_PATHWAY	**hsa00510:N-Glycan biosynthesis**	9.15E-03
KEGG_PATHWAY	**hsa00564:Glycerophospholipid metabolism**	9.77E-03
GOTERM_BP_DIRECT	**GO:0006024~glycosaminoglycan biosynthetic process**	1.05E-02
GOTERM_BP_DIRECT	**GO:0019276~UDP-N-acetylgalactosamine metabolic process**	1.10E-02
GOTERM_BP_DIRECT	**GO:0050651~dermatan sulfate proteoglycan biosynthetic process**	1.10E-02
GOTERM_BP_DIRECT	**GO:0006094~gluconeogenesis**	1.15E-02
GOTERM_BP_DIRECT	**GO:0043647~inositol phosphate metabolic process**	1.30E-02
GOTERM_BP_DIRECT	**GO:0006670~sphingosine metabolic process**	1.47E-02
GOTERM_BP_DIRECT	**GO:0043456~regulation of pentose-phosphate shunt**	1.83E-02
KEGG_PATHWAY	**hsa00534:Glycosaminoglycan biosynthesis - heparan sulfate / heparin**	1.88E-02
KEGG_PATHWAY	**hsa00062:Fatty acid elongation**	2.04E-02
GOTERM_BP_DIRECT	**GO:0001933~negative regulation of protein phosphorylation**	2.13E-02
GOTERM_BP_DIRECT	GO:0008152~metabolic process	2.42E-02
GOTERM_BP_DIRECT	GO:0016485~protein processing	2.91E-02
GOTERM_BP_DIRECT	**GO:0006002~fructose 6-phosphate metabolic process**	2.92E-02
GOTERM_BP_DIRECT	**GO:0050650~chondroitin sulfate proteoglycan biosynthetic process**	2.92E-02
KEGG_PATHWAY	**hsa00051:Fructose and mannose metabolism**	3.24E-02
GOTERM_BP_DIRECT	GO:0008354~germ cell migration	3.28E-02
GOTERM_BP_DIRECT	**GO:0006000~fructose metabolic process**	3.63E-02

GOTERM_BP_DIRECT	**GO:0036065~fucosylation**	3.99E-02
GOTERM_BP_DIRECT	GO:0046135~pyrimidine nucleoside catabolic process	3.99E-02
GOTERM_BP_DIRECT	**GO:0046855~inositol phosphate dephosphorylation**	3.99E-02
GOTERM_BP_DIRECT	GO:0002003~angiotensin maturation	3.99E-02
GOTERM_BP_DIRECT	**GO:0017187~peptidyl-glutamic acid carboxylation**	3.99E-02
GOTERM_BP_DIRECT	**GO:0045820~negative regulation of glycolytic process**	3.99E-02
GOTERM_BP_DIRECT	**GO:0030166~proteoglycan biosynthetic process**	4.34E-02
REACTOME_PATHWAY	Transport of gamma-carboxylated protein precursors from the endoplasmic reticulum to the Golgi apparatus	4.47E-02
KEGG_PATHWAY	**hsa04975:Fat digestion and absorption**	4.65E-02
GOTERM_BP_DIRECT	GO:0071456~cellular response to hypoxia	4.90E-02
REACTOME_PATHWAY	Removal of aminoterminal propeptides from gamma-carboxylated proteins	4.96E-02
REACTOME_PATHWAY	Gamma-carboxylation of protein precursors	4.96E-02

**Database**	**Enriched pathways in LUAD**	**P-Value**
GOTERM_BP_DIRECT	**GO:0006508~proteolysis**	2.73E-21
GOTERM_BP_DIRECT	GO:0015991~ATP hydrolysis coupled proton transport	7.80E-19
KEGG_PATHWAY	**hsa04610:Complement and coagulation cascades**	6.94E-18
KEGG_PATHWAY	hsa00190:Oxidative phosphorylation	1.34E-15
GOTERM_BP_DIRECT	GO:0034220~ion transmembrane transport	2.04E-14
GOTERM_BP_DIRECT	GO:0042776~mitochondrial ATP synthesis coupled proton transport	2.09E-14
REACTOME_PATHWAY	Formation of ATP by chemiosmotic coupling	1.84E-13
GOTERM_BP_DIRECT	GO:0006754~ATP biosynthetic process	4.29E-13
GOTERM_BP_DIRECT	GO:0015986~ATP synthesis coupled proton transport	4.30E-12
KEGG_PATHWAY	hsa04972:Pancreatic secretion	1.14E-11
GOTERM_BP_DIRECT	GO:0033572~transferrin transport	1.62E-10
REACTOME_PATHWAY	Ion channel transport	2.18E-10
REACTOME_PATHWAY	Insulin receptor recycling	4.10E-10
REACTOME_PATHWAY	Transferrin endocytosis and recycling	9.57E-10
GOTERM_BP_DIRECT	GO:0090383~phagosome acidification	1.78E-09
GOTERM_BP_DIRECT	GO:0015992~proton transport	1.97E-09
KEGG_PATHWAY	hsa04966:Collecting duct acid secretion	9.34E-09
GOTERM_BP_DIRECT	**GO:0042730~fibrinolysis**	2.99E-08
GOTERM_BP_DIRECT	GO:0008286~insulin receptor signaling pathway	5.46E-08
KEGG_PATHWAY	hsa05110:Vibrio cholerae infection	6.28E-08
KEGG_PATHWAY	hsa05150:Staphylococcus aureus infection	8.53E-08
REACTOME_PATHWAY	**Activation of Matrix Metalloproteinases**	1.02E-07
REACTOME_PATHWAY	**ROS, RNS production in phagocytes**	1.23E-07
REACTOME_PATHWAY	Ion transport by P-type ATPases	1.39E-07
REACTOME_PATHWAY	**Intrinsic Pathway of Fibrin Clot Formation**	3.86E-07
GOTERM_BP_DIRECT	**GO:0007597~blood coagulation, intrinsic pathway**	1.07E-06
GOTERM_BP_DIRECT	**GO:0022617~extracellular matrix disassembly**	1.11E-06
KEGG_PATHWAY	**hsa05010:Alzheimer's disease**	1.47E-06

GOTERM_BP_DIRECT	**GO:0006956~complement activation**	2.46E-06
GOTERM_BP_DIRECT	GO:1903779~regulation of cardiac conduction	4.97E-06
KEGG_PATHWAY	hsa05120:Epithelial cell signaling in Helicobacter pylori infection	6.49E-06
GOTERM_BP_DIRECT	**GO:0017187~peptidyl-glutamic acid carboxylation**	1.38E-05
REACTOME_PATHWAY	Transport of gamma-carboxylated protein precursors from the endoplasmic reticulum to the Golgi apparatus	2.86E-05
KEGG_PATHWAY	hsa05323:Rheumatoid arthritis	3.96E-05
REACTOME_PATHWAY	Removal of aminoterminal propeptides from gamma-carboxylated proteins	4.06E-05
REACTOME_PATHWAY	Gamma-carboxylation of protein precursors	4.06E-05
REACTOME_PATHWAY	Ion homeostasis	4.65E-05
KEGG_PATHWAY	hsa04721:Synaptic vesicle cycle	5.28E-05
GOTERM_BP_DIRECT	**GO:0051919~positive regulation of fibrinolysis**	1.17E-04
REACTOME_PATHWAY	**Reduction of cytosolic Ca++ levels**	1.21E-04
KEGG_PATHWAY	hsa04971:Gastric acid secretion	1.22E-04
KEGG_PATHWAY	**hsa05012:Parkinson's disease**	1.29E-04
GOTERM_BP_DIRECT	**GO:0007596~blood coagulation**	1.72E-04
GOTERM_BP_DIRECT	GO:0006465~signal peptide processing	1.83E-04
KEGG_PATHWAY	hsa04145:Phagosome	1.89E-04
KEGG_PATHWAY	**hsa05016:Huntington's disease**	1.96E-04
KEGG_PATHWAY	**hsa04974:Protein digestion and absorption**	3.41E-04
REACTOME_PATHWAY	Regulation of Insulin-like Growth Factor (IGF) transport and uptake by Insulin-like Growth Factor Binding Proteins (IGFBPs)	4.25E-04
GOTERM_BP_DIRECT	**GO:0016311~dephosphorylation**	5.91E-04
GOTERM_BP_DIRECT	**GO:0031638~zymogen activation**	6.94E-04
GOTERM_BP_DIRECT	GO:0036376~sodium ion export from cell	8.65E-04
GOTERM_BP_DIRECT	**GO:0016241~regulation of macroautophagy**	9.94E-04
GOTERM_BP_DIRECT	**GO:0006958~complement activation, classical pathway**	1.00E-03
GOTERM_BP_DIRECT	GO:0030007~cellular potassium ion homeostasis	1.26E-03
KEGG_PATHWAY	hsa04022:cGMP-PKG signaling pathway	1.46E-03
GOTERM_BP_DIRECT	GO:0010248~establishment or maintenance of transmembrane electrochemical gradient	1.49E-03
KEGG_PATHWAY	hsa01100:Metabolic pathways	1.51E-03
REACTOME_PATHWAY	**Initial triggering of complement**	1.99E-03
KEGG_PATHWAY	hsa04970:Salivary secretion	2.22E-03
GOTERM_BP_DIRECT	GO:0007586~digestion	2.80E-03
GOTERM_BP_DIRECT	GO:0006883~cellular sodium ion homeostasis	3.20E-03
REACTOME_PATHWAY	**Dissolution of Fibrin Clot**	3.74E-03
GOTERM_BP_DIRECT	GO:0010107~potassium ion import	6.90E-03
GOTERM_BP_DIRECT	**GO:0030449~regulation of complement activation**	7.90E-03
GOTERM_BP_DIRECT	**GO:0006874~cellular calcium ion homeostasis**	8.33E-03
GOTERM_BP_DIRECT	**GO:0032470~positive regulation of endoplasmic reticulum calcium ion concentration**	8.91E-03
GOTERM_BP_DIRECT	**GO:0002542~Factor XII activation**	8.91E-03
GOTERM_BP_DIRECT	GO:0045851~pH reduction	8.91E-03

GOTERM_BP_DIRECT	**GO:0035335~peptidyl-tyrosine dephosphorylation**	9.88E-03
REACTOME_PATHWAY	**Common Pathway of Fibrin Clot Formation**	1.06E-02
KEGG_PATHWAY	**hsa04020:Calcium signaling pathway**	1.22E-02
KEGG_PATHWAY	**hsa04961:Endocrine and other factor-regulated calcium reabsorption**	1.23E-02
GOTERM_BP_DIRECT	GO:0045087~innate immune response	1.24E-02
GOTERM_BP_DIRECT	**GO:1903416~response to glycoside**	1.33E-02
GOTERM_BP_DIRECT	**GO:1901660~calcium ion export**	1.33E-02
GOTERM_BP_DIRECT	GO:0044267~cellular protein metabolic process	1.58E-02
KEGG_PATHWAY	hsa04261:Adrenergic signaling in cardiomyocytes	1.61E-02
GOTERM_BP_DIRECT	**GO:0070588~calcium ion transmembrane transport**	1.62E-02
REACTOME_PATHWAY	**Synthesis of IP3 and IP4 in the cytosol**	1.69E-02
REACTOME_PATHWAY	**Regulation of Complement cascade**	1.69E-02
GOTERM_BP_DIRECT	**GO:0043647~inositol phosphate metabolic process**	1.87E-02
GOTERM_BP_DIRECT	**GO:0007598~blood coagulation, extrinsic pathway**	2.21E-02
GOTERM_BP_DIRECT	GO:0045822~negative regulation of heart contraction	3.09E-02
GOTERM_BP_DIRECT	GO:0046069~cGMP catabolic process	3.09E-02
GOTERM_BP_DIRECT	**GO:0001867~complement activation, lectin pathway**	3.09E-02
GOTERM_BP_DIRECT	GO:0006888~ER to Golgi vesicle-mediated transport	3.48E-02
GOTERM_BP_DIRECT	GO:0014909~smooth muscle cell migration	3.52E-02
GOTERM_BP_DIRECT	GO:0086036~regulation of cardiac muscle cell membrane potential	3.52E-02
REACTOME_PATHWAY	Ficolins bind to repetitive carbohydrate structures on the target cell surface	3.53E-02
REACTOME_PATHWAY	**Extrinsic Pathway of Fibrin Clot Formation**	3.53E-02
REACTOME_PATHWAY	**Alternative complement activation**	3.53E-02
KEGG_PATHWAY	**hsa04918:Thyroid hormone synthesis**	3.92E-02
GOTERM_BP_DIRECT	**GO:0031639~plasminogen activation**	3.95E-02
GOTERM_BP_DIRECT	GO:0050778~positive regulation of immune response	3.95E-02
KEGG_PATHWAY	**hsa00562:Inositol phosphate metabolism**	4.06E-02
KEGG_PATHWAY	**hsa04142:Lysosome**	4.08E-02
GOTERM_BP_DIRECT	GO:0016485~protein processing	4.12E-02
REACTOME_PATHWAY	**Lectin pathway of complement activation**	4.22E-02
GOTERM_BP_DIRECT	**GO:0051918~negative regulation of fibrinolysis**	4.38E-02
GOTERM_BP_DIRECT	**GO:0071383~cellular response to steroid hormone stimulus**	4.38E-02

**Database**	**Enriched pathways in LUSC**	**P-Value**
GOTERM_BP_DIRECT	**GO:0016311~dephosphorylation**	2.57E-13
KEGG_PATHWAY	**hsa04610:Complement and coagulation cascades**	1.08E-10
GOTERM_BP_DIRECT	**GO:0006508~proteolysis**	9.16E-09
GOTERM_BP_DIRECT	GO:0015991~ATP hydrolysis coupled proton transport	7.07E-08
GOTERM_BP_DIRECT	**GO:0006470~protein dephosphorylation**	2.19E-07
GOTERM_BP_DIRECT	**GO:0042730~fibrinolysis**	6.84E-07
GOTERM_BP_DIRECT	GO:0015986~ATP synthesis coupled proton transport	8.34E-07

REACTOME_PATHWAY	**Intrinsic Pathway of Fibrin Clot Formation**	4.70E-06
GOTERM_BP_DIRECT	**GO:0035335~peptidyl-tyrosine dephosphorylation**	2.11E-05
GOTERM_BP_DIRECT	**GO:0007597~blood coagulation, intrinsic pathway**	2.92E-05
GOTERM_BP_DIRECT	**GO:0051919~positive regulation of fibrinolysis**	6.76E-05
GOTERM_BP_DIRECT	GO:0034220~ion transmembrane transport	7.60E-05
KEGG_PATHWAY	hsa00190:Oxidative phosphorylation	8.08E-05
GOTERM_BP_DIRECT	**GO:0031638~zymogen activation**	4.01E-04
GOTERM_BP_DIRECT	**GO:0017187~peptidyl-glutamic acid carboxylation**	6.11E-04
KEGG_PATHWAY	hsa05150:Staphylococcus aureus infection	8.60E-04
REACTOME_PATHWAY	Transport of gamma-carboxylated protein precursors from the endoplasmic reticulum to the Golgi apparatus	9.63E-04
REACTOME_PATHWAY	Gamma-carboxylation of protein precursors	1.20E-03
REACTOME_PATHWAY	Removal of aminoterminal propeptides from gamma-carboxylated proteins	1.20E-03
GOTERM_BP_DIRECT	**GO:0006672~ceramide metabolic process**	1.49E-03
GOTERM_BP_DIRECT	**GO:0006629~lipid metabolic process**	1.98E-03
GOTERM_BP_DIRECT	GO:0042776~mitochondrial ATP synthesis coupled proton transport	2.28E-03
GOTERM_BP_DIRECT	**GO:0006956~complement activation**	3.21E-03
GOTERM_BP_DIRECT	GO:0006465~signal peptide processing	3.23E-03
REACTOME_PATHWAY	Ion transport by P-type ATPases	3.33E-03
GOTERM_BP_DIRECT	**GO:0007596~blood coagulation**	3.51E-03
REACTOME_PATHWAY	Formation of ATP by chemiosmotic coupling	3.97E-03
GOTERM_BP_DIRECT	GO:0006754~ATP biosynthetic process	4.33E-03
GOTERM_BP_DIRECT	GO:0035774~positive regulation of insulin secretion involved in cellular response to glucose stimulus	4.33E-03
GOTERM_BP_DIRECT	GO:0033572~transferrin transport	6.27E-03
GOTERM_BP_DIRECT	**GO:0002542~Factor XII activation**	6.78E-03
KEGG_PATHWAY	**hsa00564:Glycerophospholipid metabolism**	6.80E-03
REACTOME_PATHWAY	Ion channel transport	7.02E-03
KEGG_PATHWAY	hsa05110:Vibrio cholerae infection	8.11E-03
REACTOME_PATHWAY	Insulin receptor recycling	8.21E-03
REACTOME_PATHWAY	Transferrin endocytosis and recycling	1.02E-02
GOTERM_BP_DIRECT	**GO:0043647~inositol phosphate metabolic process**	1.11E-02
GOTERM_BP_DIRECT	GO:0015992~proton transport	1.20E-02
REACTOME_PATHWAY	**Activation of Matrix Metalloproteinases**	1.30E-02
GOTERM_BP_DIRECT	**GO:0002084~protein depalmitoylation**	1.35E-02
GOTERM_BP_DIRECT	**GO:0006670~sphingosine metabolic process**	1.35E-02
GOTERM_BP_DIRECT	GO:1903779~regulation of cardiac conduction	1.55E-02
KEGG_PATHWAY	**hsa04142:Lysosome**	1.56E-02
GOTERM_BP_DIRECT	**GO:0043456~regulation of pentose-phosphate shunt**	1.69E-02
KEGG_PATHWAY	hsa04966:Collecting duct acid secretion	1.94E-02
GOTERM_BP_DIRECT	**GO:0014068~positive regulation of phosphatidylinositol 3-kinase signaling**	2.05E-02
KEGG_PATHWAY	hsa01100:Metabolic pathways	2.56E-02
GOTERM_BP_DIRECT	**GO:0022617~extracellular matrix disassembly**	2.75E-02

GOTERM_BP_DIRECT	GO:0008286~insulin receptor signaling pathway	2.88E-02
GOTERM_BP_DIRECT	**GO:0031639~plasminogen activation**	3.01E-02
GOTERM_BP_DIRECT	GO:0070072~vacuolar proton-transporting V-type ATPase complex assembly	3.34E-02
GOTERM_BP_DIRECT	**GO:0051918~negative regulation of fibrinolysis**	3.34E-02
GOTERM_BP_DIRECT	**GO:0016042~lipid catabolic process**	3.38E-02
REACTOME_PATHWAY	Ion homeostasis	3.52E-02
GOTERM_BP_DIRECT	GO:0060081~membrane hyperpolarization	3.67E-02
GOTERM_BP_DIRECT	**GO:0046855~inositol phosphate dephosphorylation**	3.67E-02
KEGG_PATHWAY	hsa04972:Pancreatic secretion	3.79E-02
REACTOME_PATHWAY	**Activation of C3 and C5**	4.16E-02
GOTERM_BP_DIRECT	**GO:0016540~protein autoprocessing**	4.33E-02
GOTERM_BP_DIRECT	**GO:0006958~complement activation, classical pathway**	4.45E-02
KEGG_PATHWAY	**hsa05010:Alzheimer's disease**	4.47E-02
GOTERM_BP_DIRECT	GO:0007035~vacuolar acidification	4.65E-02
GOTERM_BP_DIRECT	**GO:0030194~positive regulation of blood coagulation**	4.65E-02

**Database**	**Enriched pathways in PRAD**	**P-Value**
GOTERM_BP_DIRECT	**GO:0006508~proteolysis**	1.25E-20
KEGG_PATHWAY	**hsa04610:Complement and coagulation cascades**	1.86E-19
GOTERM_BP_DIRECT	**GO:0016311~dephosphorylation**	2.43E-10
GOTERM_BP_DIRECT	**GO:0006486~protein glycosylation**	2.85E-09
REACTOME_PATHWAY	**Activation of Matrix Metalloproteinases**	5.63E-08
KEGG_PATHWAY	hsa01100:Metabolic pathways	6.71E-08
KEGG_PATHWAY	hsa05150:Staphylococcus aureus infection	8.53E-08
GOTERM_BP_DIRECT	**GO:0017187~peptidyl-glutamic acid carboxylation**	1.46E-07
REACTOME_PATHWAY	Transport of gamma-carboxylated protein precursors from the endoplasmic reticulum to the Golgi apparatus	1.98E-07
REACTOME_PATHWAY	**Intrinsic Pathway of Fibrin Clot Formation**	2.36E-07
REACTOME_PATHWAY	Gamma-carboxylation of protein precursors	3.29E-07
REACTOME_PATHWAY	Removal of aminoterminal propeptides from gamma-carboxylated proteins	3.29E-07
GOTERM_BP_DIRECT	**GO:0007597~blood coagulation, intrinsic pathway**	1.32E-06
GOTERM_BP_DIRECT	**GO:0022617~extracellular matrix disassembly**	1.50E-06
GOTERM_BP_DIRECT	**GO:0007596~blood coagulation**	2.40E-06
GOTERM_BP_DIRECT	**GO:0042730~fibrinolysis**	2.56E-06
GOTERM_BP_DIRECT	**GO:0006956~complement activation**	3.34E-06
GOTERM_BP_DIRECT	GO:0006465~signal peptide processing	5.32E-06
KEGG_PATHWAY	**hsa00601:Glycosphingolipid biosynthesis - lacto and neolacto series**	6.93E-06
KEGG_PATHWAY	**hsa00533:Glycosaminoglycan biosynthesis - keratan sulfate**	1.54E-05
GOTERM_BP_DIRECT	**GO:0009312~oligosaccharide biosynthetic process**	1.61E-05
GOTERM_BP_DIRECT	**GO:0005975~carbohydrate metabolic process**	1.76E-05
GOTERM_BP_DIRECT	**GO:0015012~heparan sulfate proteoglycan biosynthetic process**	6.50E-05
GOTERM_BP_DIRECT	**GO:0043647~inositol phosphate metabolic process**	6.94E-05

REACTOME_PATHWAY	**N-Glycan antennae elongation**	1.12E-04
GOTERM_BP_DIRECT	**GO:0051919~positive regulation of fibrinolysis**	1.30E-04
KEGG_PATHWAY	**hsa00510:N-Glycan biosynthesis**	1.66E-04
GOTERM_BP_DIRECT	**GO:0046835~carbohydrate phosphorylation**	1.66E-04
GOTERM_BP_DIRECT	**GO:0007598~blood coagulation, extrinsic pathway**	2.17E-04
KEGG_PATHWAY	**hsa00052:Galactose metabolism**	2.74E-04
GOTERM_BP_DIRECT	**GO:0018146~keratan sulfate biosynthetic process**	3.02E-04
REACTOME_PATHWAY	Regulation of Insulin-like Growth Factor (IGF) transport and uptake by Insulin-like Growth Factor Binding Proteins (IGFBPs)	3.19E-04
REACTOME_PATHWAY	**Extrinsic Pathway of Fibrin Clot Formation**	4.10E-04
GOTERM_BP_DIRECT	**GO:0006002~fructose 6-phosphate metabolic process**	6.01E-04
KEGG_PATHWAY	**hsa04070:Phosphatidylinositol signaling system**	6.08E-04
REACTOME_PATHWAY	**Keratan sulfate biosynthesis**	7.62E-04
REACTOME_PATHWAY	**Synthesis of IP3 and IP4 in the cytosol**	7.62E-04
GOTERM_BP_DIRECT	**GO:0031639~plasminogen activation**	7.70E-04
GOTERM_BP_DIRECT	**GO:0006487~protein N-linked glycosylation**	8.11E-04
KEGG_PATHWAY	**hsa00562:Inositol phosphate metabolism**	9.42E-04
GOTERM_BP_DIRECT	**GO:0006024~glycosaminoglycan biosynthetic process**	1.01E-03
GOTERM_BP_DIRECT	**GO:0006958~complement activation, classical pathway**	1.22E-03
KEGG_PATHWAY	**hsa01200:Carbon metabolism**	1.29E-03
REACTOME_PATHWAY	**Initial triggering of complement**	1.39E-03
KEGG_PATHWAY	**hsa00514:Other types of O-glycan biosynthesis**	1.59E-03
KEGG_PATHWAY	**hsa00534:Glycosaminoglycan biosynthesis - heparan sulfate / heparin**	2.06E-03
REACTOME_PATHWAY	**Dissolution of Fibrin Clot**	3.10E-03
GOTERM_BP_DIRECT	GO:0007586~digestion	3.25E-03
GOTERM_BP_DIRECT	GO:0016485~protein processing	4.73E-03
KEGG_PATHWAY	**hsa00051:Fructose and mannose metabolism**	4.74E-03
GOTERM_BP_DIRECT	**GO:0046856~phosphatidylinositol dephosphorylation**	5.64E-03
KEGG_PATHWAY	**hsa00010:Glycolysis / Gluconeogenesis**	5.67E-03
GOTERM_BP_DIRECT	GO:0006888~ER to Golgi vesicle-mediated transport	6.86E-03
GOTERM_BP_DIRECT	**GO:0030449~regulation of complement activati**on	8.73E-03
REACTOME_PATHWAY	**Common Pathway of Fibrin Clot Formation**	8.83E-03
GOTERM_BP_DIRECT	**GO:0006001~fructose catabolic process**	9.39E-03
GOTERM_BP_DIRECT	**GO:0005986~sucrose biosynthetic process**	9.39E-03
GOTERM_BP_DIRECT	**GO:0033692~cellular polysaccharide biosynthetic process**	9.39E-03
GOTERM_BP_DIRECT	GO:0006096~glycolytic process	1.11E-02
GOTERM_BP_DIRECT	**GO:0035335~peptidyl-tyrosine dephosphorylation**	1.14E-02
GOTERM_BP_DIRECT	**GO:0016310~phosphorylation**	1.17E-02
REACTOME_PATHWAY	**Regulation of Complement cascade**	1.41E-02
KEGG_PATHWAY	hsa04974:Protein digestion and absorption	1.46E-02
KEGG_PATHWAY	hsa04972:Pancreatic secretion	1.75E-02
GOTERM_BP_DIRECT	GO:0044267~cellular protein metabolic process	1.82E-02
GOTERM_BP_DIRECT	**GO:0006670~sphingosine metabolic process**	1.87E-02

GOTERM_BP_DIRECT	**GO:0015014~heparan sulfate proteoglycan biosynthetic process, polysaccharide chain biosynthetic process**	1.87E-02
REACTOME_PATHWAY	Synthesis of PIPs at the plasma membrane	2.15E-02
GOTERM_BP_DIRECT	**GO:0006470~protein dephosphorylation**	2.16E-02
KEGG_PATHWAY	hsa01130:Biosynthesis of antibiotics	2.60E-02
GOTERM_BP_DIRECT	**GO:0006661~phosphatidylinositol biosynthetic process**	3.05E-02
REACTOME_PATHWAY	Ficolins bind to repetitive carbohydrate structures on the target cell surface	3.21E-02
REACTOME_PATHWAY	**Alternative complement activation**	3.21E-02
GOTERM_BP_DIRECT	**GO:0030388~fructose 1,6-bisphosphate metabolic process**	3.25E-02
GOTERM_BP_DIRECT	**GO:0001867~complement activation, lectin pathway**	3.25E-02
GOTERM_BP_DIRECT	**GO:0006110~regulation of glycolytic process**	3.25E-02
KEGG_PATHWAY	hsa05202:Transcriptional misregulation in cancer	3.34E-02
GOTERM_BP_DIRECT	GO:0014909~smooth muscle cell migration	3.70E-02
GOTERM_BP_DIRECT	GO:0046626~regulation of insulin receptor signaling pathway	3.70E-02
REACTOME_PATHWAY	**Lectin pathway of complement activation**	3.84E-02
KEGG_PATHWAY	**hsa00030:Pentose phosphate pathway**	3.84E-02
GOTERM_BP_DIRECT	GO:0050778~positive regulation of immune response	4.16E-02
GOTERM_BP_DIRECT	**GO:0031638~zymogen activation**	4.16E-02
GOTERM_BP_DIRECT	**GO:0042355~L-fucose catabolic process**	4.61E-02
GOTERM_BP_DIRECT	**GO:0051156~glucose 6-phosphate metabolic process**	4.61E-02
GOTERM_BP_DIRECT	**GO:0006000~fructose metabolic process**	4.61E-02
GOTERM_BP_DIRECT	**GO:0051918~negative regulation of fibrinolysis**	4.61E-02
KEGG_PATHWAY	**hsa00500:Starch and sucrose metabolism**	4.86E-02

**Database**	**Enriched pathways in STAD**	**P-Value**
KEGG_PATHWAY	hsa01100:Metabolic pathways	2.20E-21
GOTERM_BP_DIRECT	**GO:0016310~phosphorylation**	5.68E-21
KEGG_PATHWAY	**hsa04070:Phosphatidylinositol signaling system**	1.24E-12
GOTERM_BP_DIRECT	**GO:0016311~dephosphorylation**	5.25E-12
GOTERM_BP_DIRECT	**GO:0046835~carbohydrate phosphorylation**	6.68E-10
KEGG_PATHWAY	**hsa00562:Inositol phosphate metabolism**	3.04E-09
GOTERM_BP_DIRECT	**GO:0006661~phosphatidylinositol biosynthetic process**	2.36E-07
GOTERM_BP_DIRECT	**GO:0005975~carbohydrate metabolic process**	1.17E-06
GOTERM_BP_DIRECT	**GO:0043647~inositol phosphate metabolic process**	2.20E-06
KEGG_PATHWAY	hsa01130:Biosynthesis of antibiotics	1.40E-05
GOTERM_BP_DIRECT	GO:0006096~glycolytic process	1.62E-05
GOTERM_BP_DIRECT	**GO:0046834~lipid phosphorylation**	1.90E-05
KEGG_PATHWAY	hsa01200:Carbon metabolism	2.53E-05
KEGG_PATHWAY	**hsa00561:Glycerolipid metabolism**	3.28E-05
GOTERM_BP_DIRECT	**GO:0015012~heparan sulfate proteoglycan biosynthetic process**	5.79E-05
KEGG_PATHWAY	**hsa00010:Glycolysis / Gluconeogenesis**	7.50E-05
REACTOME_PATHWAY	**Activation of Matrix Metalloproteinases**	7.90E-05
GOTERM_BP_DIRECT	**GO:0046856~phosphatidylinositol dephosphorylation**	1.68E-04

GOTERM_BP_DIRECT	**GO:0009157~deoxyribonucleoside monophosphate biosynthetic process**	2.00E-04
GOTERM_BP_DIRECT	**GO:0061624~fructose catabolic process to hydroxyacetone phosphate and glyceraldehyde-3-phosphate**	2.00E-04
KEGG_PATHWAY	**hsa00760:Nicotinate and nicotinamide metabolism**	2.39E-04
REACTOME_PATHWAY	Synthesis of PIPs at the Golgi membrane	2.53E-04
KEGG_PATHWAY	**hsa00051:Fructose and mannose metabolism**	3.53E-04
GOTERM_BP_DIRECT	GO:0007205~protein kinase C-activating G-protein coupled receptor signaling pathway	3.66E-04
GOTERM_BP_DIRECT	**GO:0022617~extracellular matrix disassembly**	3.88E-04
GOTERM_BP_DIRECT	**GO:0030388~fructose 1,6-bisphosphate metabolic process**	4.18E-04
REACTOME_PATHWAY	Effects of PIP2 hydrolysis	4.68E-04
REACTOME_PATHWAY	**Fructose catabolism**	4.83E-04
GOTERM_BP_DIRECT	**GO:0046854~phosphatidylinositol phosphorylation**	8.68E-04
GOTERM_BP_DIRECT	**GO:0006024~glycosaminoglycan biosynthetic process**	9.01E-04
GOTERM_BP_DIRECT	**GO:0035335~peptidyl-tyrosine dephosphorylation**	1.05E-03
GOTERM_BP_DIRECT	**GO:0006071~glycerol metabolic process**	1.08E-03
GOTERM_BP_DIRECT	**GO:0036092~phosphatidylinositol-3-phosphate biosynthetic process**	1.41E-03
GOTERM_BP_DIRECT	**GO:0006486~protein glycosylation**	1.72E-03
GOTERM_BP_DIRECT	**GO:0030168~platelet activation**	1.83E-03
GOTERM_BP_DIRECT	**GO:0009165~nucleotide biosynthetic process**	2.04E-03
KEGG_PATHWAY	**hsa00534:Glycosaminoglycan biosynthesis - heparan sulfate / heparin**	2.06E-03
REACTOME_PATHWAY	Synthesis of IP2, IP, and Ins in the cytosol	2.58E-03
KEGG_PATHWAY	hsa00230:Purine metabolism	2.71E-03
KEGG_PATHWAY	hsa05230:Central carbon metabolism in cancer	4.82E-03
GOTERM_BP_DIRECT	GO:0061621~canonical glycolysis	6.12E-03
GOTERM_BP_DIRECT	**GO:0033692~cellular polysaccharide biosynthetic process**	9.03E-03
GOTERM_BP_DIRECT	GO:0046104~thymidine metabolic process	9.03E-03
GOTERM_BP_DIRECT	**GO:0046167~glycerol-3-phosphate biosynthetic process**	1.35E-02
KEGG_PATHWAY	hsa00450:Selenocompound metabolism	1.40E-02
REACTOME_PATHWAY	**Synthesis of IP3 and IP4 in the cytosol**	1.64E-02
GOTERM_BP_DIRECT	**GO:0006670~sphingosine metabolic process**	1.80E-02
GOTERM_BP_DIRECT	**GO:0015014~heparan sulfate proteoglycan biosynthetic process, polysaccharide chain biosynthetic process**	1.80E-02
GOTERM_BP_DIRECT	**GO:0006470~protein dephosphorylation**	1.95E-02
REACTOME_PATHWAY	Glycolysis	2.12E-02
GOTERM_BP_DIRECT	**GO:0043456~regulation of pentose-phosphate shunt**	2.24E-02
KEGG_PATHWAY	hsa00240:Pyrimidine metabolism	2.30E-02
GOTERM_BP_DIRECT	GO:0001501~skeletal system development	2.42E-02
REACTOME_PATHWAY	Synthesis of PIPs at the plasma membrane	2.51E-02
GOTERM_BP_DIRECT	**GO:0006508~proteolysis**	2.56E-02
GOTERM_BP_DIRECT	**GO:0050428~3'-phosphoadenosine 5'-phosphosulfate biosynthetic process**	2.69E-02
GOTERM_BP_DIRECT	**GO:0032958~inositol phosphate biosynthetic process**	2.69E-02
GOTERM_BP_DIRECT	**GO:0019563~glycerol catabolic process**	2.69E-02

REACTOME_PATHWAY	Metabolism of ingested H2SeO4 and H2SeO3 into H2Se	2.79E-02
REACTOME_PATHWAY	Synthesis of IPs in the nucleus	2.79E-02
GOTERM_BP_DIRECT	GO:0051289~protein homotetramerization	3.02E-02
GOTERM_BP_DIRECT	**GO:0030259~lipid glycosylation**	3.13E-02
GOTERM_BP_DIRECT	**GO:0046486~glycerolipid metabolic process**	3.13E-02
GOTERM_BP_DIRECT	GO:0007586~digestion	3.31E-02
REACTOME_PATHWAY	Glucose metabolism	3.48E-02
GOTERM_BP_DIRECT	**GO:0006002~fructose 6-phosphate metabolic process**	3.56E-02
GOTERM_BP_DIRECT	**GO:0000103~sulfate assimilation**	3.56E-02
GOTERM_BP_DIRECT	GO:0035556~intracellular signal transduction	3.58E-02
GOTERM_BP_DIRECT	GO:0032868~response to insulin	3.70E-02
GOTERM_BP_DIRECT	**GO:0006006~glucose metabolic process**	3.70E-02
KEGG_PATHWAY	**hsa00030:Pentose phosphate pathway**	3.84E-02
GOTERM_BP_DIRECT	GO:0050778~positive regulation of immune response	4.00E-02
KEGG_PATHWAY	**hsa00052:Galactose metabolism**	4.09E-02
REACTOME_PATHWAY	Transport and synthesis of PAPS	4.16E-02
GOTERM_BP_DIRECT	GO:0016485~protein processing	4.22E-02
GOTERM_BP_DIRECT	**GO:0006000~fructose metabolic process**	4.44E-02
GOTERM_BP_DIRECT	**GO:0051156~glucose 6-phosphate metabolic process**	4.44E-02
GOTERM_BP_DIRECT	**GO:0009247~glycolipid biosynthetic process**	4.44E-02
KEGG_PATHWAY	**hsa00500:Starch and sucrose metabolism**	4.86E-02
GOTERM_BP_DIRECT	**GO:0046855~inositol phosphate dephosphorylation**	4.87E-02
GOTERM_BP_DIRECT	**GO:0035970~peptidyl-threonine dephosphorylation**	4.87E-02
GOTERM_BP_DIRECT	GO:0045820~negative regulation of glycolytic process	4.87E-02
GOTERM_BP_DIRECT	GO:0002003~angiotensin maturation	4.87E-02
GOTERM_BP_DIRECT	GO:0046135~pyrimidine nucleoside catabolic process	4.87E-02
GOTERM_BP_DIRECT	GO:0050728~negative regulation of inflammatory response	4.98E-02

**Database**	**Enriched pathways in THCA**	**P-Value**
GOTERM_BP_DIRECT	**GO:0006508~proteolysis**	2.05E-19
KEGG_PATHWAY	**hsa04610:Complement and coagulation cascades**	2.38E-19
GOTERM_BP_DIRECT	**GO:0006470~protein dephosphorylation**	6.26E-13
GOTERM_BP_DIRECT	**GO:0035335~peptidyl-tyrosine dephosphorylation**	8.70E-10
REACTOME_PATHWAY	**Activation of Matrix Metalloproteinases**	2.86E-09
REACTOME_PATHWAY	**Intrinsic Pathway of Fibrin Clot Formation**	7.99E-09
GOTERM_BP_DIRECT	**GO:0007597~blood coagulation, intrinsic pathway**	1.66E-08
GOTERM_BP_DIRECT	**GO:0042730~fibrinolysis**	3.89E-08
GOTERM_BP_DIRECT	**GO:0022617~extracellular matrix disassembly**	6.55E-08
GOTERM_BP_DIRECT	**GO:0017187~peptidyl-glutamic acid carboxylation**	1.46E-07
REACTOME_PATHWAY	Transport of gamma-carboxylated protein precursors from the endoplasmic reticulum to the Golgi apparatus	3.13E-07
GOTERM_BP_DIRECT	**GO:0051919~positive regulation of fibrinolysis**	4.00E-07
REACTOME_PATHWAY	Gamma-carboxylation of protein precursors	5.19E-07

REACTOME_PATHWAY	Removal of aminoterminal propeptides from gamma-carboxylated proteins	5.19E-07
KEGG_PATHWAY	hsa05150:Staphylococcus aureus infection	1.62E-06
GOTERM_BP_DIRECT	**GO:0007596~blood coagulation**	2.40E-06
GOTERM_BP_DIRECT	**GO:0016311~dephosphorylation**	3.12E-06
GOTERM_BP_DIRECT	**GO:0046835~carbohydrate phosphorylation**	3.75E-06
GOTERM_BP_DIRECT	GO:0006465~signal peptide processing	5.32E-06
GOTERM_BP_DIRECT	**GO:0031638~zymogen activation**	8.25E-06
GOTERM_BP_DIRECT	**GO:0006956~complement activation**	5.54E-05
REACTOME_PATHWAY	**Synthesis of IP2, IP, and Ins in the cytosol**	5.82E-05
GOTERM_BP_DIRECT	**GO:0043647~inositol phosphate metabolic process**	6.94E-05
GOTERM_BP_DIRECT	**GO:0030148~sphingolipid biosynthetic process**	7.54E-05
KEGG_PATHWAY	**hsa00600:Sphingolipid metabolism**	1.45E-04
GOTERM_BP_DIRECT	**GO:0006486~protein glycosylation**	1.91E-04
REACTOME_PATHWAY	**Sphingolipid de novo biosynthesis**	2.11E-04
GOTERM_BP_DIRECT	**GO:0007598~blood coagulation, extrinsic pathway**	2.17E-04
GOTERM_BP_DIRECT	GO:0007586~digestion	2.19E-04
GOTERM_BP_DIRECT	GO:0016485~protein processing	3.66E-04
REACTOME_PATHWAY	Regulation of Insulin-like Growth Factor (IGF) transport and uptake by Insulin-like Growth Factor Binding Proteins (IGFBPs)	4.45E-04
REACTOME_PATHWAY	**Extrinsic Pathway of Fibrin Clot Formation**	5.14E-04
KEGG_PATHWAY	**hsa00533:Glycosaminoglycan biosynthesis - keratan sulfate**	5.16E-04
GOTERM_BP_DIRECT	**GO:0031639~plasminogen activation**	7.70E-04
GOTERM_BP_DIRECT	**GO:0046855~inositol phosphate dephosphorylation**	1.17E-03
GOTERM_BP_DIRECT	**GO:0046839~phospholipid dephosphorylation**	1.17E-03
GOTERM_BP_DIRECT	**GO:0006958~complement activation, classical pathway**	1.22E-03
GOTERM_BP_DIRECT	**GO:0030194~positive regulation of blood coagulation**	1.92E-03
KEGG_PATHWAY	**hsa00601:Glycosphingolipid biosynthesis - lacto and neolacto series**	2.70E-03
GOTERM_BP_DIRECT	**GO:0006796~phosphate-containing compound metabolic process**	3.18E-03
REACTOME_PATHWAY	**Dissolution of Fibrin Clot**	3.86E-03
KEGG_PATHWAY	hsa01100:Metabolic pathways	4.19E-03
GOTERM_BP_DIRECT	GO:0009235~cobalamin metabolic process	4.33E-03
REACTOME_PATHWAY	**N-Glycan antennae elongation**	5.15E-03
GOTERM_BP_DIRECT	**GO:0046856~phosphatidylinositol dephosphorylation**	5.64E-03
GOTERM_BP_DIRECT	**GO:0000188~inactivation of MAPK activity**	6.11E-03
GOTERM_BP_DIRECT	GO:0006888~ER to Golgi vesicle-mediated transport	6.86E-03
KEGG_PATHWAY	**hsa00562:Inositol phosphate metabolism**	7.30E-03
GOTERM_BP_DIRECT	**GO:0018146~keratan sulfate biosynthetic process**	7.63E-03
GOTERM_BP_DIRECT	**GO:0030449~regulation of complement activation**	8.73E-03
GOTERM_BP_DIRECT	**GO:0005975~carbohydrate metabolic process**	9.16E-03
GOTERM_BP_DIRECT	**GO:0002542~Factor XII activation**	9.39E-03
GOTERM_BP_DIRECT	**GO:0033692~cellular polysaccharide biosynthetic process**	9.39E-03
REACTOME_PATHWAY	Cobalamin (Cbl, vitamin B12) transport and metabolism	1.00E-02
REACTOME_PATHWAY	**Common Pathway of Fibrin Clot Formation**	1.10E-02

GOTERM_BP_DIRECT	**GO:0016310~phosphorylation**	1.17E-02
KEGG_PATHWAY	**hsa00565:Ether lipid metabolism**	1.27E-02
REACTOME_PATHWAY	DARPP-32 events	1.51E-02
KEGG_PATHWAY	**hsa00520:Amino sugar and nucleotide sugar metabolism**	1.52E-02
GOTERM_BP_DIRECT	GO:1901215~negative regulation of neuron death	1.52E-02
REACTOME_PATHWAY	**Initial triggering of complement**	1.71E-02
REACTOME_PATHWAY	**Keratan sulfate biosynthesis**	1.74E-02
REACTOME_PATHWAY	**Regulation of Complement cascade**	1.74E-02
GOTERM_BP_DIRECT	GO:0044267~cellular protein metabolic process	1.82E-02
GOTERM_BP_DIRECT	**GO:0006670~sphingosine metabolic process**	1.87E-02
GOTERM_BP_DIRECT	**GO:0006021~inositol biosynthetic process**	1.87E-02
GOTERM_BP_DIRECT	**GO:0015014~heparan sulfate proteoglycan biosynthetic process, polysaccharide chain biosynthetic process**	1.87E-02
KEGG_PATHWAY	**hsa00564:Glycerophospholipid metabolism**	1.97E-02
KEGG_PATHWAY	**hsa04070:Phosphatidylinositol signaling system**	2.18E-02
GOTERM_BP_DIRECT	**GO:0043456~regulation of pentose-phosphate shunt**	2.33E-02
GOTERM_BP_DIRECT	**GO:0006644~phospholipid metabolic process**	2.67E-02
REACTOME_PATHWAY	Negative regulation of MAPK pathway	3.10E-02
GOTERM_BP_DIRECT	**GO:0006020~inositol metabolic process**	3.25E-02
REACTOME_PATHWAY	**Alternative complement activation**	3.58E-02
GOTERM_BP_DIRECT	GO:0033173~calcineurin-NFAT signaling cascade	3.70E-02
GOTERM_BP_DIRECT	GO:0014909~smooth muscle cell migration	3.70E-02
GOTERM_BP_DIRECT	GO:0008354~germ cell migration	4.16E-02
GOTERM_BP_DIRECT	**GO:0006012~galactose metabolic process**	4.16E-02
GOTERM_BP_DIRECT	GO:0050778~positive regulation of immune response	4.16E-02
GOTERM_BP_DIRECT	**GO:0051918~negative regulation of fibrinolysis**	4.61E-02
GOTERM_BP_DIRECT	GO:0051533~positive regulation of NFAT protein import into nucleus	4.61E-02
